# Ultrafast Multilevel Switching and Synaptic Behavior in a Planar Quantum Topological Memristor

**DOI:** 10.1002/advs.202520413

**Published:** 2026-01-30

**Authors:** Mamoon Ur Rashid, Usman Safder, Sobia Ali Khan, Anh‐Tuan Pham, Muhammad Sheeraz, Nguyen‐Hoang Dang, Duy Le Thanh, Zeeshan Tahir, Faisal Maqbool, Koo‐Hyun Chung, Sunglae Cho, Jungdae Kim, Yong Soo Kim

**Affiliations:** ^1^ Department of Semiconductor Engineering and Energy Harvest‐Storage Research Center University of Ulsan Ulsan South Korea; ^2^ School of Chemical and Bioprocess Engineering, Engineering and Materials Science Centre University College Dublin (UCD), Belfield Campus Dublin Ireland; ^3^ New Energy Materials Research Institute Korea Institute of Energy Technology (KENTECH) Naju South Korea; ^4^ Division of Chemical and Material Metrology Korea Research Institute of Standards and Science (KRISS) Daejeon South Korea; ^5^ Department of Mechanical Engineering University of Ulsan Ulsan South Korea; ^6^ Department of Physics and Chemistry Daegu Gyeongbuk Institute of Science and Technology (DGIST) Daegu South Korea

**Keywords:** energy efficient, image recognition, planar memristor, topological insulator, ultrafast‐multilevel switching

## Abstract

The rapid increase in data driven by analytics and Internet of Things demands innovation in both device architecture and materials to meet the growing need for fast and efficient computing. Here we report an ultrafast planar quantum topological memristor (PQTM), comprised of bismuth‐telluride (Bi_2_Te_3_) thin film transferred onto pre‐patterned electrodes. Owing to the planar architecture, the device connects both electrodes to the surface states of Bi_2_Te_3,_ offering a platform to directly benefit from the characteristic features of topological surface states, such as low‐dissipation and scattering‐resistant channels essential for ultrafast‐ and efficient‐charge transport. Pertinently, PQTM presents a forming‐free bipolar‐resistive switching behavior with an ultrafast‐switching ∼15 ± 5 ns and low‐energy consumption ∼14.5 nJ, which is a record high among the topological insulator‐based memristors. Moreover, the endurance evaluation over 10^3^ consecutive DC‐switching cycles demonstrates superior stability in both high and low resistive states, while the retention tests display an excellent longevity of ∼10^5^ s, signifying reliable non‐volatile operation. Finally, PQTM reproducibility is established via comparison with 24 other devices, presenting multilevel resistive switching exhibiting both digital and analog switching modes together with long‐term potentiation, depression, and persistent image‐recognition performance, corroborated via 1D‐convolutional layers with four LeNet models. Thus, our work emphasizes the critical role of device architecture in harnessing material properties for advanced‐memory and neuromorphic applications.

## Graphical abstract

Dry‐transferred Bi_2_Te_3_ layers enable a planar quantum topological memristor framework. In‐plane topological surface states facilitate ultrafast & low‐power operations. Coexisting analog and digital modes support current‐controlled multilevel states. PQTM exhibits 10^5^ s retention, 10^3^ cycles endurance, and reproducibility across 24 devices. Achieved synaptic functions and image recognition for neuromorphic memory.

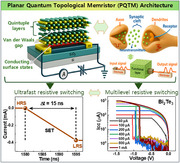

## 1 Introduction

Advancements in next‐generation electronics require fast and energy‐efficient multifunctional devices that combine perception, memory, and computation in a single architecture [1, 2]. This approach draws inspiration from the human brain, where biological synapses inherently integrate data processing and storage functions, enabling rapid multitasking capability with exceptionally low power consumption and high speed [3−5]. The neural system performs synaptic operations at ∼10 fJ with total power consumption under 20 W across ∼10^15^ synapses, thereby driving the development of artificial neuromorphic systems aiming at ultralow power and ultrafast speed parallelism [1, 6]. In‐memory computing, capable of processing and storing information simultaneously, offers a promising solution to the limitations and bottlenecks inherent in conventional von Neumann architecture‐based computing systems [7]. Among various memory and neuromorphic computing technologies, memristors are regarded as a fundamental device architecture [8]. They have attracted considerable interest due to their simple structure, non‐volatility, and, most importantly, their ability to emulate biological synaptic behavior, making them ideal candidates for hardware‐based neural networks [9, 10]. The thin functional layer sandwiched between the top and bottom electrodes in these state‐of‐the‐art vertical memristor architectures enables enhanced scalability and high‐density array integration, making them compatible with complementary metal‐oxide‐semiconductor technology [11, 12]. However, these devices often face significant challenges, including interfacial complexity, poor control over deposition layers and switching sites, fabrication‐induced stress, and difficulty in probing the functional layer matrix to observe the exact structural changes during operation [13−16]. Despite extensive research, the underlying mechanisms of resistive switching remain unclear and open to debate [17, 18]. In contrast, planar memristor architecture, where the functional layer lies laterally between two coplanar electrodes, offers greater versatility for multi‐terminal configurations by enabling the addition of extra electrodes or optical stimuli. This design allows for precise control over electrical properties, lower intrinsic capacitance, pulse‐tunable characteristics, facile interface engineering, and an enhanced dynamic response [12, 16, 19]. Moreover, the absence of multilayer stacking in planar devices facilitates easier integration with 2D/3D layered materials and enables in‐situ surface characterization during operation [12, 16, 20, 21].

Typically memristors have been widely investigated using diverse functional materials such as oxides and their heterojunctions [10, 22, 23, 24], perovskites [25, 26], quantum dots [27, 28], 2D layered materials [29, 30] and many more, for applications like resistance random access memory [31], phase change memory [32], synaptic memory [33, 34], neuromorphic computing [35], and sensing neuron based memristors [36]. Recent planar devices have incorporated 2D materials like MoS_2_ [37]_,_ graphene oxide [38], black phosphorus [39], and organic molecules [40], many of which exploit van der Waals interfaces to realize low‐power switching. Nevertheless, realizing ultrafast switching time, stable endurance, minimal variability, robust synaptic plasticity, and image recognition capability in planar memristor architectures remains an open challenge [41, 42]. Moreover, topological insulators (TIs) have been rarely explored as functional layers in vertical memristor architectures [43−45], while their application in planar configurations remains unexplored. To address this gap, a comprehensive study on the integration of 3D TI materials into planar memristors is necessary. TIs represent a unique class of quantum materials that are neither conventional conductors nor true insulators; these materials behave as insulators in the bulk while hosting topologically protected, gapless conducting states on their surfaces or edges. These surface states are robust against defects, impurities, and scattering, while they exhibit high carrier mobility, enabling ultrafast and energy‐efficient switching [44−47]. To effectively harness surface‐state conduction, an in‐plane (planar) device architecture is considered more suitable than conventional out‐of‐plane (vertical) configurations, as it enables both electrodes to interact with the same topological surfaces [48]. The detailed conceptual illustration is mentioned in Note S1 and Figure S1. Thus, planar architecture enables effective utilization of non‐dissipative edge channels and high surface mobility, thereby realizing an ultrafast and energy‐efficient quantum memristor.

In this study, we present a planar quantum topological memristor (PQTM) architecture utilizing a layered single‐crystal Bi_2_Te_3_ synthesized through a temperature gradient method. This planar architecture has been fabricated via a dry transfer technique using a homebuilt micromanipulator over prepatterned Au electrodes to enable direct interaction with topological surface states (TSS) responsible for high‐speed switching. The cumulative effect of planar configuration combines with low dissipative edge states, revealing forming‐free bipolar resistive switching with both abrupt and gradual transitions, exhibiting fast (ns) and energy‐efficient (nJ) resistive switching, stable endurance over 10^3^ repeated DC cycles with a resistance ratio of ∼2 × 10^2^ maintained for 10^5^ s. Reproducibility was validated across 24 devices, all demonstrating consistent switching behavior with stable high resistive state (HRS) and low resistive state (LRS). Finally, multilevel resistive switching (MRS) with both digital and analog resistive switching capabilities, along with synaptic characteristics evidenced by long‐term potentiation (LTP) and long‐term depression (LTD) analysis, was demonstrated. High image recognition performance further highlights the potential of PQTM for advanced sensing and neuromorphic computing applications.

## 2 Results and Discussion

**FIGURE 1:**
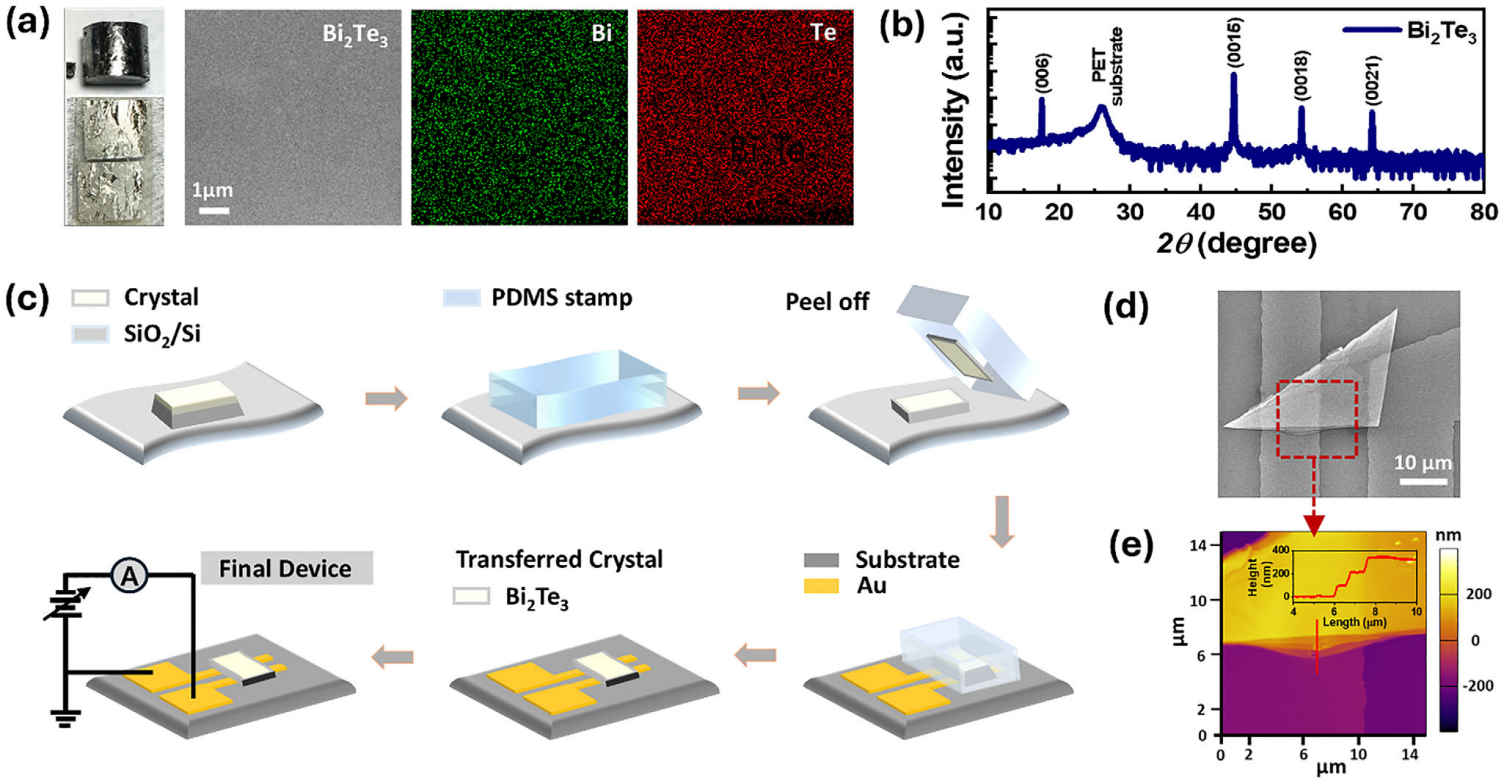
Characterization of Bi_2_Te_3_ single crystals and the device fabrication process. (a) Left panel: Digital images represent the Bi_2_Te_3_ single crystals; Right panel: SEM and EDX images acquired from the surface of the exfoliated Bi_2_Te_3,_ indicating a smooth morphology and uniform elemental composition. (b) XRD pattern of Bi_2_Te_3_ transferred onto PET substrates, showing only (00*l*) family of planes, confirming the single‐crystal nature. (c) Stepwise schematic of the Bi_2_Te_3_ transfer process using a PDMS stamp, presenting the stacking of thin film onto pre‐patterned Au electrodes to form the planar memristor architecture. (d) SEM image of the as‐transferred Bi_2_Te_3_ indicates the successful bridging between Au electrodes with clean interfacial contact. (e) AFM height map of the Bi_2_Te_3_ surface shows a clean and featureless morphology. The inset presents a step‐like height profile, ensuring the layered structure.

The left panel of Figure 1a corresponds to the digital images of the bulk and exfoliated Bi_2_Te_3_ synthesized via the temperature gradient method, as shown in Note S2 and Figure S2_._ Subsequently, the right panel represents the SEM image and the corresponding EDS mappings of the as‐exfoliated Bi_2_Te_3_. The mapping reveals the uniform distribution of the constituent Bi (green) and Te (red) elements across the surface and the desired stoichiometry as shown in Figure S3 [49, 50]. Figure 1b presents the XRD pattern of an exfoliated thin Bi_2_Te_3_ film on a polyethylene terephthalate (PET) substrate. The *θ*‐2*θ* scans acquired over a 2*θ* scale reveal the high single‐crystalline quality with the crystal growth oriented along the (00*l*) diffraction plane. The XRD peaks indexed with (006), (0015), (0018), and (0021) peaks without the formation of any secondary peak, well match the rhombohedral structural symmetry with a space group of *R*3*m* [51, 52].

Figure 1c schematically presents the fabrication of PQTM devices. Firstly, the already exfoliated Bi_2_Te_3_ was transferred to a SiO_2_/Si substrate. Next, a PDMS stamp is used to pick up a thin layer of Bi_2_Te_3_, followed by a subsequent transfer to a target substrate coated with pre‐patterned Au electrodes using a micromanipulator (Figure S4a) to realize a planar memristor device. A micromanipulator is employed in the dry transfer process to enable precise positioning and manipulation of microscale materials. Dry mechanical transfer offers superior control and preserves material quality in contrast to wet transfer methods, which often compromise crystal integrity due to chemical exposure. Moreover, to ensure the successful release from the PDMS stamp onto the target substrate, a stepwise heat treatment is performed as depicted in Figure S4b. Finally, a bias is applied to the electrodes for electrical characterization. Figure 1d shows an SEM image of the final device. The image confirms clean contact between the as‐transferred Bi_2_Te_3_ and the Au electrode with sharp boundaries, demonstrating the successful transfer and fabrication of a planar memristor device architecture. Figure 1e presents the 2D‐AFM image of PQTM. The image clearly reveals the layered structure of Bi_2_Te_3_ with a corresponding height profile as shown in the inset.

**FIGURE 2:**
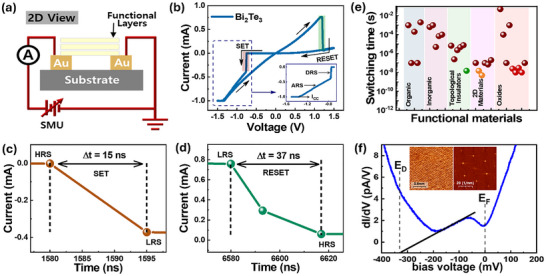
Ultrafast switching of Bi_2_Te_3_ planar memristor. (a) 2D schematic illustrations of the planar device, where a Bi_2_Te_3_ functional layer bridges the Au electrodes. (b) *I–V* characteristics showing the forming‐free bipolar resistive switching behavior with clear SET and RESET transitions. The inset highlights the zoomed SET region to clearly distinguish different resistive switching regimes, such as the digital resistive switching (DRS), and analog resistive switching (ARS) under current compliance (*I*_cc_). (c, d) Current vs time plots present the ultrafast SET and RESET transitions with a switching time of ∼15 ± 5 ns, and ∼37 ± 3 ns, respectively. (e) Benchmark plot comparing the switching speed of Bi_2_Te_3_ topological insulator with other functional materials. (f) d*I*/d*V* tunneling spectrum of the Bi_2_Te_3_ surface, characterizing the topological surface states. The inset shows an atomic resolution STM topography with its corresponding FFT pattern.

Unlike the conventional 3D vertical memristor, the planar architecture is categorized as a 2D device. Figure 2a illustrates the 2D schematic view of the Bi_2_Te_3_‐based planar memristor fabricated via a bottom‐up approach, connected to the SMU for electrical analysis. The *I–V* curves demonstrate a forming‐free and bipolar resistive switching behavior as shown in Figure 2b. Interestingly, PQTM exhibits both the digital (sharp) resistive switching (DRS) and analog (gradual) resistive switching (ARS) characteristics, rendering it suitable for multipurpose applications. In extenso, DRS is initiated at a low threshold voltage (*V*_SET_) approximately −0.7 V, followed by a transition to ARS until the device completely switches to LRS, wherein the device remains in the ON state till the threshold for the RESET voltage (*V*_RESET_) is exceeded. The transition from sharp to gradual switching (inset) at the inflection point *V*_SET_ (*I* = 0.37 mA, *R* = 2 kΩ) during the SET process suggests quantum conductance (∼500 µS), a key indicator of the coexistence of DRS and ARS [53, 54]. Besides, it is important to mention that a compliance current (*I*_cc_) of −1.0 mA is applied during the operation to prevent the hard breakdown of PQTM. Additionally, as shown in Figure 2c,d, our device achieves an ultrafast DC resistive switching performance for both SET (∼15 ± 5 ns) and RESET (∼37 ± 3 ns), respectively. Moreover, the ultrafast‐switching time is also validated from the transient current response under a 300 ns voltage pulse, as manifested in Figure S5. Interestingly, these values are comparable to the reported nanosecond range functional materials such as oxides [55−63], 2D materials [64−69], organics [70−74], inorganics [75−79], and TIs [44, 45] as shown in the comparison plot (Figure 2e). The chart reveals that several 2D materials (orange) and metal oxides (red) also exhibit fast resistive switching in the nanosecond range. However, it is important to note that the demonstrated switching time (green: ∼15 ± 5 ns) of the PQTM is the fastest among the TIs‐based memristors reported till date. This superior performance is attributed to the planar architecture of the device capable of effectively utilizing the non‐dissipative state available at the surface of the topological insulator (Bi_2_Te_3_), offering scattering‐resistant and energy‐efficient conduction channels crucial for the realization of ultrafast resistive switching devices [44, 46, 80]. To verify the presence of the TSS in our Bi_2_Te_3_ samples, we performed scanning tunneling microscopy/spectroscopy (STM/S) and compared the results with the well‐established study by Alpichshev et al. [81]. As shown in Figure 2f, our d*I*/d*V* spectrum exhibits a pronounced V‐shaped density of states, where the Dirac point *E*_D_ is identified at approximately −330 meV via linear fitting. This spectral profile, including the relative position of the *E*_D_ with respect to the Fermi level *E*_F,_ closely matches the reported STS characteristics of TSS [81]. This close correspondence confirms the presence of TSS in our samples. Consequently, the planar device geometry exploits these TSS to facilitate rapid lateral charge transport under applied bias, thereby reducing the effective switching barrier and enabling forming‐free, high‐resistive switching.

**FIGURE 3:**
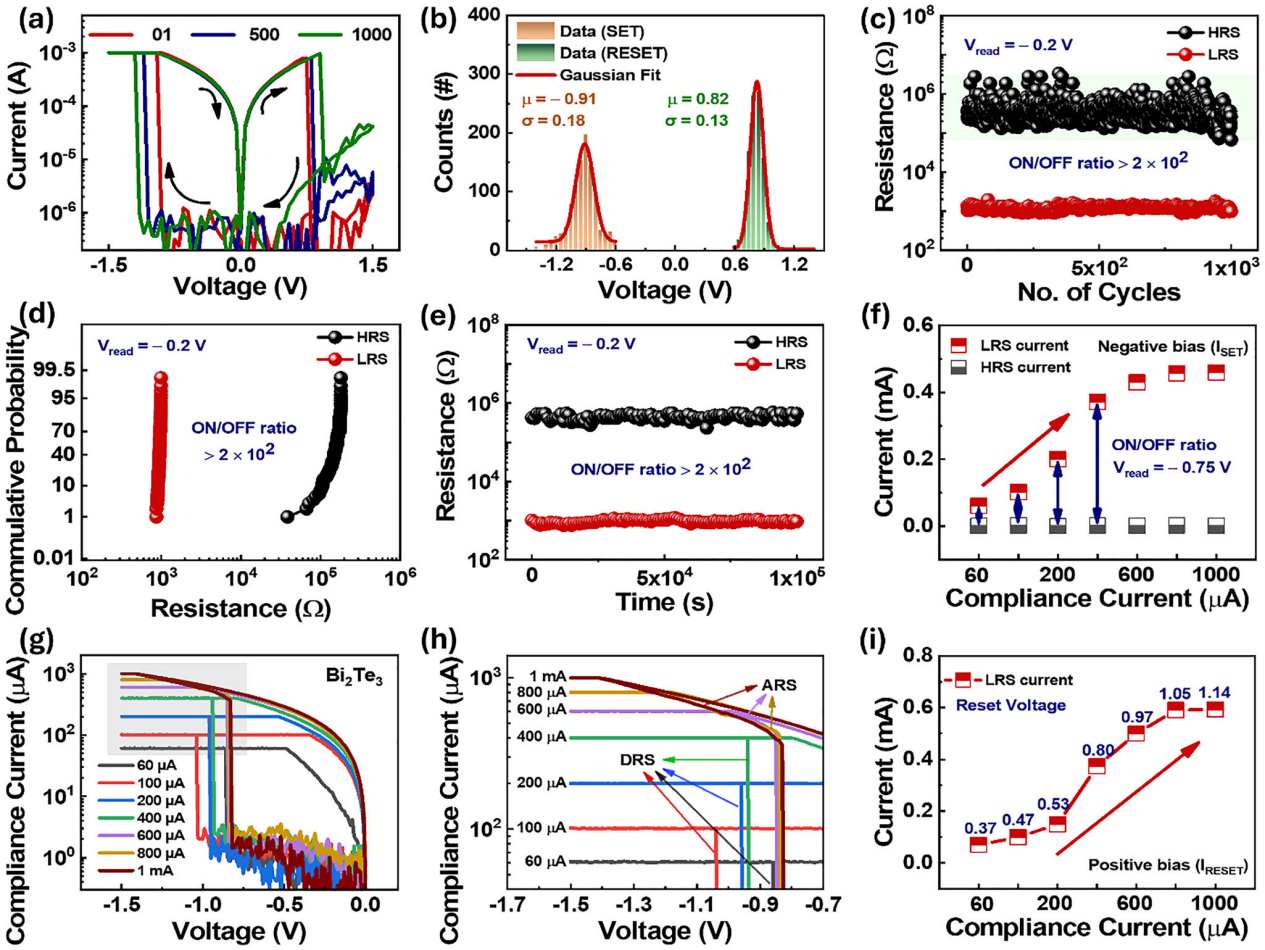
Electrical performance, switching statistics, stability, and multilevel resistive switching (MRS) of planar quantum topological memristor (PQTM). (a) Logarithmic *I–V* curves manifest a nearly uniform performance, signifying the stable bipolar resistive switching behavior of PQTM. (b) The Gaussian‐fitted voltage profile over 1000 repeated runs for SET and RESET transitions displays a narrow variability. (c) DC endurance test over 1000 consecutive switching cycles at *V*_read_ = −0.2 V demonstrates a stable ON/OFF ratio exceeding 2 × 10^2^, confirming the reliable operational stability of our device. (d) Cumulative probability distributions of the high and low resistance states over 1000 switching cycles exhibit steep and non‐overlapping curves, indicating narrow variability and high cycle‐to‐cycle stability. (e) The retention plot measured at a read voltage of −0.2 V over an extended period of 10^5^ s reveals the temporal stability of PQTM. (f) MRS tuned by varying *I*_cc_ modulates the high and low resistance state current levels and ON/OFF ratio under negative bias switching. (g) The *I–V* response in the *I*_cc_ range (60 µA to 1 mA) demonstrates a controllable switching window reflecting a current‐limited resistive behavior. (h) The *I_cc_* dependent identification of analog and digital switching reveals the role of *I*_cc_ in governing the switching dynamics. (i) Evolution of LRS current at the RESET threshold voltage under varying *I*_cc_ at positive bias demonstrates the impact of *I*_cc_ on LRS current modulation.

Moreover, stability and longevity are one of the challenging parts in such devices to investigate. Figure 3a presents the logarithmic *I–V* curve, revealing the device's cycle‐to‐cycle performance over 10^3^ DC switching cycles under a voltage sweep of ± 1.5 V across the Au electrodes with HRS and LRS in micro‐ and milli‐ampere range, respectively. This indicates the stable switching behavior observed at both polarities ensures the long‐term reliability of the PQTM. Variations observed in the threshold voltage during SET and RESET operations are quantified in Figure 3b using Gaussian fitting, yielding mean value (µ_SET_) = –0.91 V (µ_RESET_ = 0.82 V) with standard deviations (σ_SET_) = 0.18 V (σ_RESET_ = 0.13 V), indicating a good switching uniformity. Figure 3c presents the endurance performance, evaluated by extracting the HRS and LRS over 10^3^ consecutive DC cycles (Figure S6) at read voltage (*V*_read_) of −0.2 V. The device exhibits a stable ON/OFF resistance ratio exceeding 2 × 10^2^, with minor fluctuations observed in the HRS while the LRS remains highly stable. To validate the statistical stability of the resistive states, the cumulative probability distribution of the HRS and LRS values is calculated, as presented in Figure 3d. The steep and non‐overlapping cumulative curves indicate narrow distributions and low cycle‐to‐cycle variation across all 1000 switching cycles. The LRS values are clustered around ∼10^3^ Ω, while the HRS values are distributed around 10^5^ Ω. This statistical evaluation reinforces the robustness of the resistance states and confirms a similar ON/OFF ratio at *V*_read_ of −0.2 V, consistent with the endurance results in Figure 3c. Such statistical consistency is essential to quantify the device‐to‐cycle uniformity, highlighting the potential of our PQTM for memory and synaptic applications. In addition, to explain the reason behind the switching stability, the conduction mechanism was explored in the Ln(*I*) vs *V* ^1/2^ fitting plots with maximum R^2^ proving Schottky emission‐type conduction [82], presented in Figure S7. When bias is applied, it drives Au ion migration into the active layer Bi_2_Te_3_, creating dynamic trap states which help to change in conductance similar to synaptic weight transformations in biological synapses. To validate the ion migration‐based mechanism, EDX analysis has been conducted in both devices, pristine and operational, where Au presence in operational devices supports our claim very well. Moreover, to understand the facilitation of Au diffusion, we examined the reported structure of Bi‐Te, which exhibits weak bounding and a large Te atomic size [83, 84], establishing a reconfigurable trap network that enables gradual conductance transitions through combined Au migration and topological surface conductance mimicking synaptic plasticity. The time‐dependent characteristic of PQTM was examined through a retention test, as shown in Figure 3e. To assess the non‐volatile retention capability, a time‐dependent resistance measurement was performed at *V*_read_ = −0.2 V. Both resistance states were monitored over a period of 10^5^ s to confirm long‐term stability and retention, assuring an ON/OFF ratio matching well with endurance data (Figure 3c). Furthermore, the device‐to‐device reproducibility was investigated across 24 independently fabricated devices using similar experimental conditions over 50 (first 10 devices) and 200 (from device 11 to 24) DC switching cycles, as shown in Figure S8. The endurance performance extracted under the same *V*_read_ = −0.2 V, demonstrating relatively consistent switching and high device‐to‐device reliability to be implemented in large‐scale applications. In addition, the MRS characteristics were investigated under the same applied voltage by varying the compliance current (*I*_cc_) at negative bias, as shown in Figure 3f,g. Figure 3f demonstrates the current values at *V*_read_ = −0.75 V, extracted from *I–V* cycles (Figure 3g) under seven compliance levels (ranging from 60 µA to 1 mA). The result reveals seven distinct LRS currents corresponding to *I*_cc_ = 60 µA, 100 µA, 200 µA, 400 µA, 600 µA, 800 µA, and 1 mA owing to the increase in the respective current of LRS while the HRS remains nearly constant. The progressive increase in LRS conductance with higher *I*_cc_ enhances the ON/OFF ratio, indicating improved device performance at higher compliance levels. Figure 3h presents the semilogarithmic *I–V* plots acquired from the highlighted region (gray color) in Figure 3g, reaffirming that only DRS occur at *I*_cc_ ≤ 400 µA, whereas both DRS and ARS are observed at *I*_cc_ > 400 µA, consistent with the behavior studied in Figure 2b. Furthermore, Figure 3i demonstrates that an increase in *I*_cc_ leads to a corresponding rise in the maximum RESET current (*I*_RESET_). This behavior is associated with a reduction in LRS resistance, likely due to enhanced carrier mobility within the TSS of Bi_2_Te_3_ under higher compliance conditions.

**FIGURE 4:**
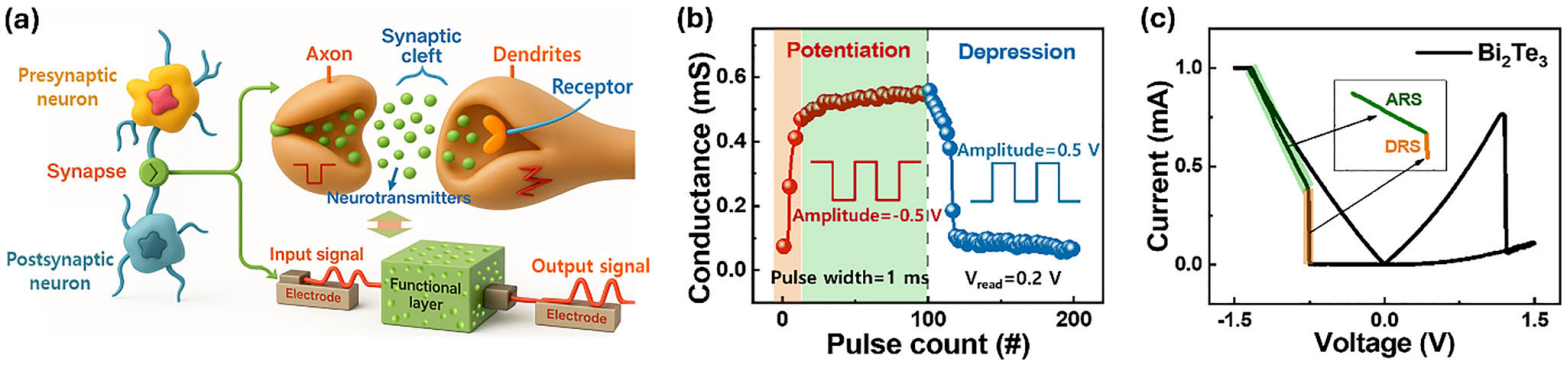
Synaptic functionality of planar quantum topological memristor (PQTM). (a) Schematic illustration of an artificial synapse mimicking biological signal transmission between presynaptic and postsynaptic neurons via a functional layer that processes input and output signals. (b) Emulation of synaptic plasticity via conductance modulation under 200 consecutive pulses induces an abrupt/gradual increase and decrease in conductance, demonstrating long‐term potentiation and long‐term depression. (c) *I*–*V* characteristics showing coexistence of analog and digital resistive switching in a PQTM. The inset highlights the transition paths associated with analog and digital switching modes under bipolar bias.

Neuromorphic activity relies on neurons and synapses that mimic brain function through highly parallel interconnections, forming the basis of neuromorphic hardware and computation [85]. To explore the synaptic capabilities of PQTM, we extended our investigation to core synaptic behaviors (LTP and LTD) by applying voltage pulses with millisecond‐range widths and intervals to assess the device's image recognition potential. Figure 4a illustrates a schematic of a biological synapse, where a presynaptic neuron connects to a postsynaptic neuron via a synaptic junction. In such systems, electrical signals are converted into chemical signals at the synaptic cleft and then back into electrical signals in the postsynaptic neuron. Specifically, action potentials (input signal) travel along axons from a presynaptic neuron, triggering vesicles to release neurotransmitters into the synaptic cleft, where receptors on the dendrite of a postsynaptic neuron generate a new action potential (output signal) and vice versa. Analogously, our device replicates this neuron‐synapse‐neuron framework using a metal/functional‐layer/metal architecture [86]. Synaptic learning and memory functions are emulated via modulation of synaptic weight, controlled by programmed presynaptic and postsynaptic voltage spikes [87]. In PQTM, two Au electrodes serve as presynaptic and postsynaptic terminals, while the Bi_2_Te_3_ functional layer enables carrier transport that mimics neurotransmitter dynamics. Therefore, to induce synaptic behavior, we applied constant square voltage pulses to one Au electrode while grounding the other. As shown in Figure 4b, consecutive 200 voltage pulses (±0.5 V) were applied to monitor conductance modulation. Negative pulses increase conductance, while positive pulses decrease it, consistent with the device's *I*–*V* behavior shown in Figure 2b. Note that the extracted conductance in Figure 4b represents the overall device conductance performance, rather than the intrinsic conductivity of the Bi_2_Te_3_ film. Moreover, the trend observed across 200 pulses demonstrates the fundamental characteristics of synaptic potentiation (increasing conductance) and depression (decreasing conductance), where a change in conductance represents synaptic weight. Upon applying 100 negative voltage pulses with a 1 ms pulse width and interval, the conductance rises from 0.07 mS to 0.56 mS, indicating LTP. Conversely, 100 positive voltage pulses decreased conductance from 0.57 mS to 0.07 mS, signifying LTD. The PQTM repeatedly replicates LTP and LTD behavior, reaffirming its applicability for long‐term operation in synaptic applications.

In general, the device response can be modulated on a cycle‐to‐cycle basis depending on the number of stimulation pulses, indicating that stable and uniform potentiation and depression occur irrespective of bias polarity and effectively emulate synaptic weight modulation and memory reinforcement in an electronic synapse. Moreover, we found that the LTP curve shows a two‐phase weight change: (i) a rapid conductance increase during the first 15 pulses (orange‐shaded region), followed by (ii) a gradual increase over the next 85 pulses (green‐shaded region). Such two‐step potentiation behavior closely resembles the abrupt (DRS) and gradual (ARS) transition observed in typical DC *I–V* cycles, as seen in Figure 4c, consistent with the trends shown in Figures 2b and 3h. Such dual switching characteristics (DRS and ARS) observed in both DC and pulse‐based data highlight the potential of PQTM for multipurpose applications, enabling efficient memory storage as well as synaptic emulation. To evaluate device performance relative to the functional layer thicknesses, we further investigated Bi_2_Te_3_ films ranging from 184.4 nm to 1320 nm across five different devices (Figure S9). As summarized in Table S1, all devices exhibit nearly identical performances in terms of stability, multilevel switching, switching time, and synaptic capability. This thickness‐independence behavior reaffirms that the active switching pathway is confined to the surface or electrode interface rather than the bulk. These results further support a surface‐assisted switching, consistent with transport facilitated by TSS in PQTMs (Figure 2f). In addition, the energy consumption of PQTM during synaptic operations was calculated using the standard relation [88]:

(1) E=V×I×t

where *V* = 0.5 V is the programming pulse amplitude, *I* is the measured current per pulse, and *t* = 1 ms is the fixed pulse width. The average energy consumption per synaptic event was estimated to be ∼14.5 nJ, while the corresponding average power consumption at read voltage of 0.2 V was ∼5.8 µW (Note S3). These results manifest the device's ultra‐low energy operation, which is compatible with biological synapses and superior to many conventional CMOS‐based artificial synaptic devices among TI_S_. This exceptional performance arises from the synergistic effect of topologically protected, non‐dissipative edge states and low‐scattering conduction channels with minimal energy loss. Consequently, PTQM demonstrates strong potential as a highly energy‐efficient platform for next‐generation neuromorphic computing applications.

**FIGURE 5:**
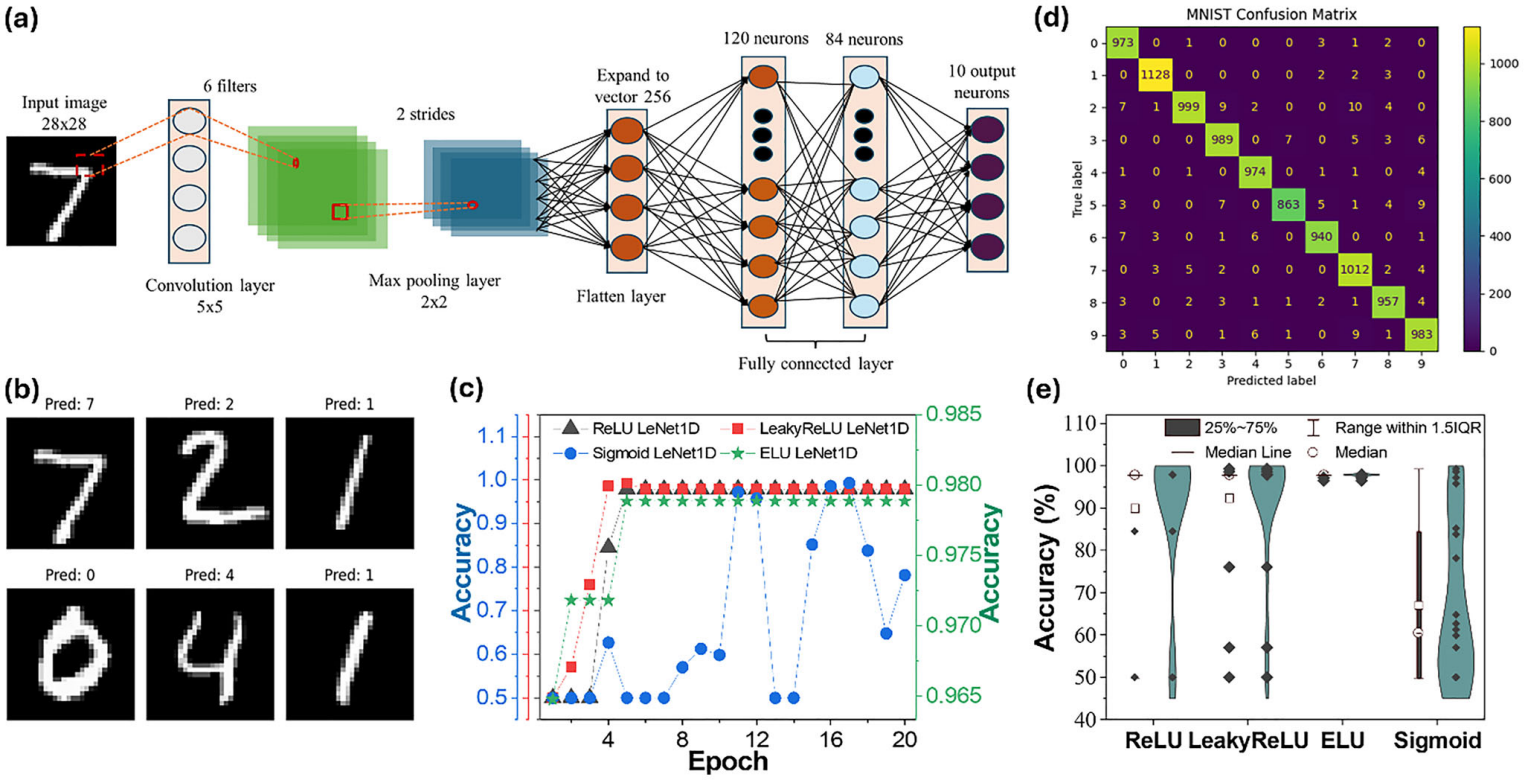
Comprehensive evaluation of neuromorphic LeNet1D variants. (a) Schematic of the neuromorphic LeNet1D pipeline: 1D convolutional encoding of potentiation/depression inputs followed by two convolutional layers, a fully connected classifier, and an output neuron. (b) MNIST test predictions reveal six randomly selected digits with inferred labels. (c) Epoch‐wise training accuracy curves for four activation functions (ReLU, LeakyReLU, ELU, Sigmoid) in the neuromorphic device, annotated with final accuracies. (d) Confusion matrix from software‐trained 2D LeNet, demonstrating per‐class recognition performance. (e) Violin‐boxplot of per‐epoch accuracy distributions across the four activations, illustrating median, interquartile range, and full distribution of training stability.

While the PQTM experimentally demonstrates synaptic behaviors such as LTP/LTD, the neuromorphic classification results presented in Figure 5 were obtained through device‐aware software simulations. The benchmark Modified National Institute of Standards and Technology (MNIST) digit recognition and LeNet‐based training were performed offline in Python 3.9.16 using PyTorch 2.1.0. These simulations utilized either the standard MNIST dataset or a numerically processed two‐element vector (𝑥_1_, 𝑥_2_) derived from the experimental device pulses (see Note S4 for details). The LeNet model architecture comprises two convolutional layers (6 and 16 filters, 5 × 5 kernel size) with average pooling layers in between, followed by three fully connected layers with 120, 84, and 10 neurons, respectively, corresponding to the ten digit classes, as shown in Figure 5a. Training was performed using the Adam optimizer for 20 epochs with a batch size of 32. The architecture is summarized in Table S2 and reflects the classical LeNet topology, which remains highly effective for benchmarking lightweight CNN performance on structured image datasets. The combined experiments demonstrate the adaptability of the LeNet framework for both device‐level neuromorphic pattern recognition and conventional image classification tasks. Figure 5b shows the predictions made by the trained LeNet model on the MNIST test set. The visualized digits 0, 1, 2, 4, and 7 were all correctly classified, with each prediction matching the corresponding image. This qualitative evaluation demonstrates the model's strong ability to accurately interpret diverse handwritten styles. The correctly classified samples include digits with varying orientations and stroke widths, illustrating the network's robustness against intra‐class variability. Notably, the digit “1” appears twice and is consistently predicted correctly, highlighting the model's reliability in identifying minimalistic digit forms. Such visual evidence complements the quantitative results, further affirming the network's effective feature extraction and class discrimination capabilities on real‐world handwritten inputs. Figure 5c compares the training dynamics of neuromorphic LeNet1D variants using four activation functions: ReLU, LeakyReLU (*α* = 0.1), ELU, and Sigmoid, over 20 epochs. All activations rapidly ascend from a 50% chance‐level baseline, with ReLU and LeakyReLU achieving the fastest convergence, exceeding 90% accuracy by epoch 5. ELU converges by approximately epoch 12, achieving accuracy comparable to ReLU‐based training. In contrast, the Sigmoid activation exhibits slower and unstable convergence, with accuracy fluctuating widely and remaining below 80% at epoch 20. The final average accuracies, annotated at each curve's terminus, are 85.3% (ReLU), 84.7% (LeakyReLU), 84.1% (ELU), and 81.5% (Sigmoid). These trends mirror the reference study's observation that piecewise‐linear activations, such as ReLU and LeakyReLU, yield faster, more robust, and stable convergence in hardware‐mapped neuromorphic networks, whereas smooth saturating functions like Sigmoid exhibit slower learning and lower asymptotic accuracy. The confusion matrix derived from the MNIST digit classification task utilizing a LeNet‐style CNN highlights noteworthy model performance, as most predictions closely correspond with the true labels, are shown in Figure 5d. The classification accuracy is notably high across all digit classes, especially for the digits 0, 1, 4, and 7, which show very few instances of misclassification. The model accurately identified 1128 instances of the digit ‘1’ and more than 970 instances of the digits 0, 2, 3, 4, 7, and 9, indicating its effectiveness in distinguishing digits with unique structural characteristics. Despite all this robustness, some confusion arises among visually similar digits. Specifically, 5 and 3 demonstrate reciprocal misclassification due to their overlapping loop structures in handwritten styles. Similar trends are observed between the digits 8 and 3 as well as between 9 and 4, reflecting challenges in differentiating digits characterized by closed loops or vertical symmetry. To assess the impact of activation functions on the classification stability of the LeNet‐based neuromorphic inference engine, four nonlinearities (ReLU, LeakyReLU, ELU, and Sigmoid) were evaluated over a sequence of 20 independent training epochs. The complete distribution of accuracy values is presented in Figure 5e. Each violin plot in Figure 5e represents a statistical sample size of *n* = 20 accuracy values extracted from consecutive training epochs. This distribution‐based visualization allows for a direct comparison of model stability, dispersion, and median performance across activation functions. ReLU and LeakyReLU display moderately broad distributions (standard deviation ≈ 14–16%), reflecting typical stochastic variability during early‐epoch optimization. Despite this spread, both converge to high‐accuracy regimes with medians near 97.9%, indicating consistent convergence toward optimal decision boundaries. In contrast, ELU exhibits the narrowest distribution (standard deviation <1%), with accuracy values tightly clustered around the median. This behavior suggests highly stable gradient flow and reduced epoch‐to‐epoch variability, consistent with ELU's smoother activation profile. Conversely, the Sigmoid function shows the widest distribution (standard deviation ≈ 18%), with accuracy values spanning 50–95%. This dispersion reflects the well‐known saturation problem of Sigmoid units, leading to higher sensitivity to initialization and slower convergence. These results suggest that ReLU‐family activations provide the optimal trade‐off between learning speed, final accuracy, and epoch‐to‐epoch stability in our 1D‐convolutional neuromorphic implementation. While ELU can achieve comparable performance with extended training, Sigmoid proves suboptimal. These findings also align with the reference implementation's conclusions that rectifying activations enables faster ID weight updates and robust inference in a compute‐in‐memory architecture. As a result, this research opens a new route in the world of memristor devices, demonstrating its potential in precision neural network computing.

## 3 Conclusion

In summary, high‐quality Bi_2_Te_3_ single crystals were synthesized using a custom‐built temperature‐gradient method and rigorously characterized by XRD, EDX, and AFM analyses. Using these high‐quality crystals, 2D‐PQTM were fabricated via mechanical exfoliation followed by a highly precise, controlled micromanipulator technique. The resultant PQTM exhibits forming‐free, ultrafast switching time (∼15 ± 5 ns), and energy‐efficient (∼14.5 nJ) bipolar resistive switching with an ON/OFF ratio greater than 2 × 10^2^ maintained over 10^3^ DC cycles and stable retention up to 10^5^ s. Robust device reproducibility was confirmed across 24 independent PQTMs, each showing consistent HRS and LRS. Furthermore, PQTMs demonstrate MRS under both digital and analog switching modes in DC and pulse simulations, effectively emulating key synaptic functions such as LTP and LTD. Finally, the image recognition characteristics were investigated using the four different 1DLeNet models on the MNIST data set, resulting in outstanding digit recognition performance. Thus, the PQTM configuration inherently exposes the active layer for optical stimuli, offering a promising platform for future optoelectronic and photonic neuromorphic applications.

## 4 Experimental Section

### 4.1 Materials

A high‐purity bismuth (Bi) and tellurium (Te) powders (99.9%) were purchased from Thermo Fisher Scientific. Polydimethylsiloxane (PDMS) was synthesized using silicone elastomer components (Sylgard 184A and 184B), which were obtained from Sewing Hi‐Tech Co., Ltd., while gold (Au, 99.9%) was procured from iTASCO.

### 4.2 Device Fabrication

The PQTM fabrication involves maskless photolithography (Figure S10a,b), electron beam evaporation (Figure S10c), and a micromanipulator setup (Figure S4a), using a TI single crystal obtained through the temperature gradient method (Figure S2). Initially, SiO_2_/Si substrates were cleaned sequentially with acetone, isopropyl alcohol, and deionized water for 10 min each, followed by nitrogen blow drying and soft backed at 60°C for one hour before photoresist coating for patterning via a photolithography system. After achieving a well‐defined pattern, Au electrodes (∼50 nm) were deposited using electron beam evaporation, as described in Figure S10c and our previous work [10]. The mechanical exfoliation was applied to thin down the bulk crystals onto a PDMS stamp for transfer, as explained in Figure 1c. Finally, a homebuilt micromanipulator was employed to transfer the thin layers from the PDMS surface onto the target substrate, where Au electrodes had already been patterned. Once the thin crystal layers were properly aligned with the target area, the temperature controller was activated to ensure a successful transfer (Figure S4b).

### 4.3 Characterizations

High‐quality TI single crystals (left panel: Figure 1a) were synthesized using a homemade vertical furnace configuration as illustrated in Note S2 and Figure S2. The XRD analyses were executed via a lab source (4‐circle) X‐ray diffractometer (wavelength = 1.5406 Å; Cu K*α*1, D8 Discover), manufactured by Bruker, Germany. Field emission scanning electron microscopy (FE‐SEM) and EDX elemental mapping images were captured by a JEOL JSM‐7600F microscope. AFM surface topography images were obtained using MFP‐3D, Asylum Research. LITHO Maskless model DLP (0.65″ DMD) having full‐HD resolution (1920 × 1080 px) was applied for electrode patterning. Homebuilt micromanipulator equipment facilitated the transfer of materials, while gold deposition was performed using an electron beam evaporator (SAEHAN, SHT‐CT800A‐DA). The Bi_2_Te_3_ single crystal was cleaved in situ in the STM chamber to obtain a clean surface for STM measurements. STM/S measurements were performed using a low‐temperature home‐built STM at 79 K with a base pressure of 2 × 10^−10^ Torr [89]. STM topographic images were acquired in the constant‐current mode with the bias voltage applied to the sample. Differential conductance (d*I*/d*V*) spectra were measured using a standard lock‐in technique with a modulation voltage and frequency of 10 mV and 1.4 kHz, respectively. Standard electrical characterizations were carried out with a Keithley‐2636A source measure unit (SMU), and pulse simulations were conducted using an Agilent B1500 semiconductor parameter analyzer equipped with pulse measurement units (PMUs). These measurements were performed with impedance‐matched cabling (50 Ohm) to minimize parasitic effects. The entire electrical characterization involved a step size of 0.05 V, with the one Au electrode biased and the other Au electrode held at ground potential.

## Conflicts of Interest

The authors declare no conflict of interest.

## Supporting information




**Supporting File**: advs73915‐sup‐0001‐SuppMat.docx.

## Data Availability

The data that support the findings of this study are available in the supplementary material of this article.

## References

[1] J. Yu , Y. Wang , S. Qin , et al., “Bioinspired Interactive Neuromorphic Devices,” Materials Today 60 (2022): 158–182, 10.1016/j.mattod.2022.09.012.

[2] T. Zhu , Y. Zhang , X. Wei , M. Jiang , and H. Xu , “The Rise of Two‐Dimensional Tellurium for Next‐Generation Electronics and Optoelectronics,” Frontiers of Physics 18 (2023): 33601, 10.1007/s11467-022-1231-9.

[3] Y. Cao , H. Fu , X. Fan , et al., “Advanced Design of High‐Performance Artificial Neuromorphic Electronics,” Materials Today 80 (2024): 648–680, 10.1016/j.mattod.2024.08.027.

[4] D. Strukov , G. Indiveri , J. Grollier , and S. Fusi , “Building Brain‐Inspired Computing,” Nature Communications 10 (2019): 4838, 10.3929/ethz-b-000394590. PMC6802080 31628303

[5] Y. Zhang , P. Qu , Y. Ji , et al., “A System Hierarchy for Brain‐Inspired Computing,” Nature 586 (2020): 378–384, 10.1038/s41586-020-2782-y. 33057220

[6] Z. Cao , B. Sun , G. Zhou , et al., “Memristor‐Based Neural Networks: A Bridge from Device to Artificial Intelligence,” Nanoscale Horizons 8 (2023): 716–745, 10.1039/D2NH00536K. 36946082

[7] B. Sun , S. Ranjan , G. Zhou , et al., “Multistate Resistive Switching Behaviors for Neuromorphic Computing in Memristor,” Materials Today Advances 9 (2021): 100125, 10.1016/j.mtadv.2020.100125.

[8] W. Chen , L. Song , S. Wang , et al., “Essential Characteristics of Memristors for Neuromorphic Computing,” Advanced Electronic Materials 9 (2023): 2200833, 10.1002/aelm.202200833.

[9] M. A. Zidan , J. P. Strachan , and W. D. Lu , “The Future of Electronics Based on Memristive Systems,” Nature Electronics 1 (2018): 22–29, 10.1038/s41928-017-0006-8.

[10] M. U. Rashid , S. A. Khan , F. Ghafoor , et al., “Role of Ti Interfacial Layer in the Stability of TiO_2_ Based Transparent Synaptic Device,” Current Applied Physics 64 (2024): 16–24, 10.1016/j.cap.2024.05.005.

[11] R. Waser , R. Dittmann , and G. Szot , “Redox‐Based Resistive Switching Memories—Nanoionic Mechanisms, Prospects, and Challenges,” Advanced Materials 21 (2009): 2632–2663, 10.1002/adma.200900375. 36751064

[12] K. Rajan , S. Bocchini , A. Chiappone , et al., “Spin‐Coated Silver Nanocomposite Resistive Switching Devices,” Microelectronic Engineering 168 (2017): 27–31, 10.1016/j.mee.2016.10.004.

[13] Y. Zuo , H. Lin , J. Guo , et al., “Effect of the Pressure Exerted by Probe Station Tips in the Electrical Characteristics of Memristors,” Advanced Electronic Materials 6 (2020): 1901226, 10.1002/aelm.201901226.

[14] L. Gao , Q. Ren , J. Sun , S. T. Han , and Y. Zhou , “Memristor Modeling: Challenges in Theories, Simulations, and Device Variability,” Journal of Materials Chemistry C 9 (2021): 16859–16884, 10.1039/D1TC04201G.

[15] Y. Xiao , B. Jiang , Z. Zhang , et al., “A Review of Memristor: Material and Structure Design, Device Performance, Applications and Prospects,” Science and Technology of Advanced Materials 24 (2023): 2162323, 10.1080/14686996.2022.2162323. 36872944 PMC9980037

[16] A. Kiazadeh , H. L. Gomes , A. R. D. Costa , et al., “Planar Non‐volatile Memory Based on Metal Nanoparticles,” MRS Online Proceedings Library (OPL) (2011): 1337, 10.1557/opl.2011.1126.

[17] S. Peng , F. Zhuge , X. Chen , et al., “Mechanism for Resistive Switching in an Oxide‐Based Electrochemical Metallization Memory,” Applied Physics Letters 100 (2012): 072101, 10.1063/1.3683523.

[18] R. Federico and G. Cicero , “Unveiling the Fundamental Role of Temperature in RRAM Switching Mechanism by multiscale simulations,” ACS Applied Materials & Interfaces 10 (2018): 7512–7519, 10.1021/acsami.8b00443. 29388424

[19] Q. Yu , R. Ge , J. Wen , et al., “Electric Pulse‐Tuned Piezotronic Effect for Interface Engineering,” Nature Communications 15 (2024): 4245, 10.1038/s41467-024-48451-6. PMC11102472 38762580

[20] X. Xin , L. Sun , J. Chen , et al., “Real‐time Numerical System Convertor via Two‐Dimensional WS2‐Based Memristive Device,” Frontiers in Computational Neuroscience 16 (2022): 1015945, 10.3389/fncom.2022.1015945. 36185713 PMC9517377

[21] F. Xuewei , X. Liu , and K. W. Ang , “2D Photonic Memristor beyond Graphene: Progress and Prospects,” Nanophotonics 9 (2020): 1579–1599, 10.1515/nanoph-2019-0543.

[22] J. Huang , H. Wang , G. Ma , et al., “A Flexible Nickel‐Oxide‐Based RRAM Device Prepared Using the Solution Combustion Method,” Electronics 13 (2024): 1042, 10.3390/electronics13061042.

[23] F. L. Aguirre , E. Piros , N. Kaiser , et al., “Revealing the Quantum Nature of the Voltage‐Induced Conductance Changes in Oxygen Engineered Yttrium Oxide‐based RRAM Devices,” Scientific Reports 14 (2024): 1122, 10.1038/s41598-023-49924-2. 38212346 PMC10784569

[24] Y. Cheng , J. Zhang , Y. Lin , et al., “Bioinspired Adaptive Neuron Enabled by Self‐powered Optoelectronic Memristor and Threshold Switching Memory for Neuromorphic Visual System,” Advanced Science 12 (2025): 2417461, 10.1002/advs.202417461. 40192159 PMC12165122

[25] C. Zhang , Y. Li , C. Ma , and Q. Zhang , “Recent Progress of Organic–Inorganic Hybrid Perovskites in RRAM, Artificial Synapse, and Logic Operation,” Small Science 2 (2020): 2100086, 10.1002/smsc.202100086. PMC11936007 40213539

[26] S. Dutta , S. Panchanan , J. H. Yoo , et al., “Synaptic Behavior of Iodine‐Enriched Copper‐Based Perovskite Memristors Developed Through a Sustainable Solution Approach,” Advanced Functional Materials 34 (2024): 2410810, 10.1002/adfm.202410810.

[27] G. Kim , S. Park , and S. Kim , “Quantum Dots for Resistive Switching Memory and Artificial Synapse,” Nanomaterials 14 (2024): 1575, 10.3390/nano14191575. 39404302 PMC11478683

[28] F. Yang , Z. Liu , X. Ding , Y. Li , C. Wang , and G. Shen , “Carbon‐Based Memristors for Resistive Random Access Memory and Neuromorphic Applications,” Chip 3 (2024): 100086, 10.1016/j.chip.2024.100086.

[29] S. Batool , M. Adrees , S. R. Zhang , S. T. Han , and Y. Zhou , “Novel Charm of 2D Materials Engineering in Memristor: When Electronics Encounter Layered Morphology,” Nanoscale Horizons 7 (2022): 480–507, 10.1039/D2NH00031H. 35343522

[30] Q. Zhao , Z. Xie , Y. P. Peng , et al., “Current Status and Prospects of Memristors Based on Novel 2D Materials,” Materials Horizons 7 (2020): 1495–1518, 10.1039/C9MH02033K.

[31] H. T. Tseng , T. H. Hsu , M. H. Tsai , C. Y. Huang , and C. L. Huang , “Resistive Switching Characteristics of Sol–Gel Derived La_2_Zr_2_O_7_ Thin Film for RRAM Applications,” Journal of Alloys and Compounds 899 (2022): 163294, 10.1016/j.jallcom.2021.163294.

[32] C. Y. Chen , Y. H. Feng , H. L. Lu , F. E. Chang , and J. Y. Chen , “Integration of ZnO‐Based Resistive‐Switching Memory and Ge_2_Sb_2_Te_5_‐Based Phase‐Change Memory,” ACS Applied Electronic Materials 5 (2023): 2583–2589, 10.1021/acsaelm.3c00064.

[33] A. Melianas , M. A. Kang , A. V. Mohammadi , et al., “High‐Speed Ionic Synaptic Memory Based on 2D Titanium Carbide MXene,” Advanced Functional Materials 32 (2022): 2109970, 10.1002/adfm.202109970.

[34] J. Lee , R. D. Nikam , M. Kwak , and H. Hwang , “Strategies to Improve the Synaptic Characteristics of Oxygen‐Based Electrochemical Random‐Access Memory Based on Material Parameters Optimization,” ACS Applied Materials & Interfaces 14 (2022): 13450–13457, 10.1021/acsami.1c21045. 35257578

[35] J. Jang , S. Gi , I. Yeo , et al., “A Learning‐Rate Modulable and Reliable TiO_x_ Memristor Array for Robust, Fast, and Accurate Neuromorphic Computing,” Advanced Science 9 (2022): 2201117, 10.1002/advs.202201117. 35666073 PMC9353447

[36] L. Wang , P. Zhang , Z. Gao , and D. Wen , “Artificial Tactile Sensing Neuron With Tactile Sensing Ability Based on a Chitosan Memristor,” Advanced Science 11 (2024): 2308610, 10.1002/advs.202308610. 38482740 PMC11109609

[37] X. Ji , S. Hao , K. G. Lim , S. Zhong , and R. Zhao , “Artificial Working Memory Constructed by Planar 2D Channel Memristors Enabling Brain‐Inspired Hierarchical Memory Systems,” Advanced Intelligent Systems 4 (2022): 2100119, 10.1002/aisy.202100119.

[38] C. He , J. Li , X. Wu , et al., “Tunable Electroluminescence in Planar Graphene/SiO_2_ Memristors,” Advanced Materials 25 (2013): 5593–5598, 10.1002/adma.201302447. 23922289

[39] X. Wan , Y. Yu , X. Wang , et al., “Large‐Area Black Phosphorus by Chemical Vapor Transport for Vertical and Lateral Memristor Devices,” The Journal of Physical Chemistry C 129 (2024): 526–534, 10.1021/acs.jpcc.4c07779.

[40] J. Zhao , W. Li , X. Wang , et al., “Organic Memristor Based on High Planar Cyanostilbene/Polymer Composite Films,” Chemical Research in Chinese Universities 39 (2023): 121–126, 10.1007/s40242-023-2352-6.

[41] S. Zhou , Y. Xing , Q. Xu , et al., “Planar Memristor and Artificial Synaptic Simulating Based on Two‐Dimensional Layered Tungsten Oxychloride WO_2_Cl_2_ ,” Applied Physics Letters 123 (2023): 241901, 10.1063/5.0177899.

[42] C. Zeng , L. Fan , S. Zhang , et al., “A Facile Planar Memristor Based on CsBi_4_Te_6_ with Asymmetric Metal Contacts,” Current Applied Physics 74 (2025): 61–66, 10.1016/j.cap.2025.04.003.

[43] A. M. Nawar , O. H. A. Eilkader , A. M. E. Mahalawy , and L. Aleya , “On Resistive Switching and Dielectric Spectroscopy Characteristics of Topological Insulator‐Based Heterojunction for Memory Applications,” Applied Physics A 130 (2024): 158, 10.1007/s00339-024-07292-2.

[44] D. S. Assi , H. Huang , V. Karthikeyan , et al., “Quantum Topological Neuristors for Advanced Neuromorphic Intelligent Systems,” Advanced Science 10 (2023): 2300791, 10.1002/advs.202300791. 37340871 PMC10460853

[45] D. S. Assi , H. Huang , V. Karthikeyan , V. C. S. Theja , M. M. D. Souza , and V. A. L. Roy , “Topological Quantum Switching Enabled Neuroelectronic Synaptic Modulators for Brain Computer Interface,” Advanced Materials 36 (2024): 2306254, 10.1002/adma.202306254. 38532608

[46] Z. Wan , Q. Zhang , F. Hu , et al., “Topological Insulator Optoelectronic Synapses for High‐Accuracy Binary Image Recognition using Recurrent Neural Networks,” Advanced Optical Materials 11 (2023): 2201852, 10.1002/adom.202201852.

[47] P. Roushan , J. Seo , C. V. Parker , et al., “Topological Surface States Protected from Backscattering by Chiral Spin Texture,” Nature 460 (2009): 1106–1109, 10.1038/nature08308. 19668187

[48] N. H. Tu , Y. Tanabe , Y. Satake , K. K. Huynh , and K. Tanigaki , “In‐Plane Topological p‐n Junction in the Three‐Dimensional Topological Insulator Bi_2−x_Sb_x_Te_3−y_Se_y_ ,” Nature Communications 7 (2016): 13763, 10.1038/ncomms13763. PMC5155151 27934857

[49] A. Raj , A. Kumar , R. Kumar , R. Kumar , and R. Chnadra , “Toward Enhancing the Thermoelectric Performance of PLD‐grown Single and Bilayer Bi_2_Te_3_ and Sb_2_Te_3_ Thin Films,” Journal of Materials Science: Materials in Electronics 35 (2024): 468, 10.1007/s10854-024-12207-1.

[50] N. D. Desai , V. L. Patil , S. S. Patil , and P. S. Patil , “Morphological Tuning of Bi_2_Se_3_ Thin Films for Photoelectrochemical Performance,” ChemistrySelect 7 (2022): 202202965, 10.1002/slct.202202965.

[51] B. K. Gupta , R. Sultana , S. Singh , et al., “Unexplored Photoluminescence from Bulk and Mechanically Exfoliated Few Layers of Bi_2_Te_3_ ,” Scientific Reports 8 (2018): 9205, 10.1038/s41598-018-27549-0. 29907865 PMC6004008

[52] Y. Kumar , R. Sultana , and V. P. S. Awana , “Comprehensive Analysis for the High Field Magneto‐Conductivity of Bi_2_Te_3_ Single Crystal,” Physica B: Condensed Matter 609 (2012): 412759, 10.1016/j.physb.2020.412759.

[53] L. Jiang , L. Xu , J. W. Chen , et al., “Conductance Quantization in an AgInSbTe‐based Memristor at Nanosecond Scale,” Applied Physics Letters 109 (2016): 153506, 10.1063/1.4963263.

[54] Z. Li , B. Tian , K. H. Xue , B. Wang , M. Xu , and H. Li , “Coexistence of Digital and Analog Resistive Switching With Low Operation Voltage in Oxygen‐Gradient HfO x Memristors,” IEEE Electron Device Letters 40 (2019): 1068–1071, 10.1109/LED.2019.2917935.

[55] Z. Wang , S. Joshi , S. E. Savelev , et al., “Memristors with Diffusive Dynamics as Synaptic Emulators for Neuromorphic Computing,” Nature Materials 16 (2017): 101, 10.1038/nmat4756. 27669052

[56] Y. W. Dai , L. Chen , W. Yang , et al., “Complementary Resistive Switching in Flexible RRAM Devices,” IEEE Electron Device Letters 35 (2014): 915–917, 10.1109/LED.2014.2334609.

[57] I. Gupta , A. Serb , A. Khiat , R. Zeitler , S. Vassanelli , and T. Prodromakis , “Real‐time Encoding and Compression of Neuronal Spikes by Metal‐Oxide Memristors,” Nature Communications 7 (2016): 12805, 10.1038/ncomms12805. PMC5052668 27666698

[58] L. Zhao , J. Xu , X. Shnag , X. Li , Q. Li , and S. Li , “Synaptic Memory Devices from CoO/Nb:SrTiO_3_ Junction,” Royal Society Open Science 6 (2019): 181098, 10.1098/rsos.181098. 31183114 PMC6502371

[59] H. Madan , H. T. Zhang , M. Jerry , et al., “26.5 Terahertz Electrically Triggered RF Switch on Epitaxial VO_2_‐on‐Sapphire (VOS) Wafer,” IEEE International Electron Devices Meeting (2015): 9, 10.1109/IEDM.2015.7409661.

[60] S. Kim , S. Choi , and W. Lu , “Comprehensive Physical Model of Dynamic Resistive Switching in an Oxide Memristor,” ACS Nano 8 (2014): 2369–2376, 10.1021/nn405827t. 24571386

[61] X. Yan , L. Zhang , H. Chen , et al., “Graphene Oxide Quantum Dots Based Memristors With Progressive Conduction Tuning for Artificial Synaptic Learning,” Advanced Functional Materials 28 (2018): 1803728, 10.1002/adfm.201803728.

[62] M. J. Lee , C. B. Lee , D. Lee , et al., “A Fast, High‐Endurance and Scalable Non‐Volatile Memory Device Made from Asymmetric Ta_2_O_5−x_/TaO_2−x_ Bilayer Structures,” Nature Materials 10 (2011): 625–630, 10.1038/nmat3070. 21743450

[63] Q. Wu , W. Banerjee , J. Cao , Z. Ji , L. Li , and M. Liu , “Improvement of Durability and Switching Speed by Incorporating Nanocrystals in the HfOx Based Resistive Random Access Memory Devices,” Applied Physics Letters 113 (2018): 023105, 10.1063/1.5030780.

[64] M. Wang , S. Cai , C. Pan , et al., “Robust Memristors Based on Layered Two‐Dimensional Materials,” Nature Electronics 1 (2018): 130–136, 10.1038/s41928-018-0021-4.

[65] X. Wu , R. Ge , M. Kim , D. Akinwande , and J. C. Lee , “Atomristors: Non‐Volatile Resistance Switching in 2D Monolayers,” Pan Pacific Microelectronics Symposium (2020): 1–6, 10.23919/PanPacific48324.2020.9059369.

[66] F. Zhang , H. Zhang , P. R. Shrestha , et al., “An Ultra‐Fast Multi‐Level MoTe_2_‐based RRAM,” IEEE IEDM (2018): 22–27, 10.1109/IEDM.2018.8614512.

[67] H. X. Li , Q. X. Li , F. Z. Li , et al., “Ni Single‐Atoms Based Memristors With Ultrafast Speed and Ultralong Data Retention,” Advanced Materials 36 (2024): 2308153, 10.1002/adma.202308153. 37939686

[68] R. D. Nikam and H. Hwang , “Atomic Threshold Switch Based on All‐2D Material Heterostructures with Excellent Control Over Filament Growth and Volatility,” Advanced Functional Materials 32 (2022): 2201749, 10.1002/adfm.202201749.

[69] S. Chen , M. R. Mahmoodi , Y. Shi , et al., “Wafer‐Scale Integration of Two‐Dimensional Materials in High‐Density Memristive Crossbar Arrays for Artificial Neural Networks,” Nature Electronics 3 (2020): 638–645, 10.1038/s41928-020-00473-w.

[70] Y. Y. Zhao , W. J. Sun , J. Wang , et al., “All‐Inorganic Ionic Polymer‐Based Memristor for High‐Performance and Flexible Artificial Synapse,” Advanced Functional Materials 30 (2020): 2004245, 10.1002/adfm.202004245.

[71] L. Zhou , J. Y. Mao , Y. Ren , et al., “Biological Spiking Synapse Constructed from Solution Processed Bimetal Core–Shell Nanoparticle Based Composites,” Small 14 (2018): 1800288, 10.1002/smll.201800288. 29806246

[72] L. Yiyang , T. P. Xiao , C. H. Bennett , et al., “In Situ Parallel Training of Analog Neural Network Using Electrochemical Random‐Access Memory,” Frontiers in neuroscience 15 (2021): 636127, 10.3389/fnins.2021.636127. 33897351 PMC8060477

[73] Q. Lai , L. Zhang , Z. Li , W. F. Stickle , R. S. Williams , and Y. Chen , “Ionic/Electronic Hybrid Materials Integrated in a Synaptic Transistor with Signal Processing and Learning Functions,” Advanced Materials 22 (2010): 2448, 10.1002/adma.201000282. 20446309

[74] S. T. Han , Y. Zhou , and V. A. L. Roy , “Towards the Development of Flexible Non‐Volatile Memories,” Advanced Materials 25 (2013): 5425, 10.1002/adma.201301361. 24038631

[75] W. Xu , H. Cho , Y. H. Kim , et al., “Organometal Halide Perovskite Artificial Synapses,” Advanced Materials 28 (2016): 5916–5922, 10.1002/adma.201506363. 27167384

[76] J. Di , J. Du , Z. Lin , S. F. Liu , J. Ouyang , and J. Chang , “Recent Advances in Resistive Random Access Memory Based on Lead Halide Perovskite,” InfoMat 3 (2021): 293, 10.1002/inf2.12162.

[77] T. Sakamoto , H. Sunamura , H. Kawaura , T. Hasegawa , T. Nakayama , and M. Aono , “Nanometer‐Scale Switches Using Copper Sulfide,” Applied Physics Letters 82 (2003): 3032, 10.1063/1.1572964.

[78] S. Kim , K. Heo , S. lee , et al., “Ferroelectric Polymer‐Based Artificial Synapse for Neuromorphic Computing,” Nanoscale Horizons 6 (2021): 139, 10.1039/D0NH00559B. 33367448

[79] S. Choi , S. H. Tan , Z. Li , et al., “SiGe Epitaxial Memory for Neuromorphic Computing with Reproducible High Performance Based on Engineered Dislocations,” Nature Materials 17 (2018): 335, 10.1038/s41563-017-0001-5. 29358642

[80] J. E. Moore , “The Birth of Topological Insulators,” Nature 464 (2010): 194–198, 10.1038/nature08916. 20220837

[81] Z. Alpichshev , J. G. Analytis , J. H. Chu , et al., “STM Imaging of Electronic Waves on the Surface of Bi2Te3: Topologically Protected Surface States and Hexagonal Warping Effects,” Physical Review Letters 104 (2010), 10.1103/PhysRevLett.104.016401. 20366373

[82] F. C. Chiu , “A Review on Conduction Mechanisms in Dielectric Films,” Advances in Materials Science and Engineering 1 (2014): 578168.

[83] A. Soni , Z. Yanyuan , Y. Ligen , M. K. K. Aik , M. S. Dresselhaus , and Q. Xiong , “Enhanced Thermoelectric Properties of Solution Grown Bi_2_Te_3–x_Se_x_ Nanoplatelet Composites,” Nano Letters 12 (2012): 1203–1209, 10.1021/nl2034859. 22295990

[84] Y. N. Nguyen and I. Son , “Diffusion Bonding at the Interface of Bi_2_Te_3_ Thermoelectric Modules,” Materials Chemistry and Physics 292 (2022): 126813, 10.1016/j.matchemphys.2022.126813.

[85] K. S. Ahmed and F. F. Shereif , “Neuromorphic Computing between Reality and Future Needs,” Neuromorphic Computing (2023): 110097, 10.5772/intechopen.110097.

[86] F. Gul , “Nano‐Scale Single Layer TiO_2_‐Based Artificial Synaptic Device,” Applied Nanoscience 10 (2020): 611–616, 10.1007/s13204-019-01179-y.

[87] Y. Sun , J. Li , S. Li , et al., “Advanced Synaptic Devices and Their Applications in Biomimetic Sensory Neural System,” Chip 2 (2023): 100031, 10.1016/j.chip.2022.100031.

[88] D. S. Jeong , K. M. Kim , S. Kim , B. J. Choi , and C. S. Hwang , “Memristors for Energy‐Efficient New Computing Paradigms,” Advanced Electronic Materials 2 (2016): 1600090, 10.1002/aelm.201600090.

[89] J. Kim , H. Nam , S. Qin , et al., “Compact Low Temperature Scanning Tunneling Microscope with In Situ Sample Preparation Capability,” Review of Scientific Instruments 86 (2015): 093707, 10.1063/1.4931761. 26429448

