# Supplementary material for: Ultrafast Multilevel Switching and Synaptic Behavior in a Planar Quantum Topological Memristor
Source: Adv Sci (Weinh). 2026 Jan 30;13(21):e20413. doi: 10.1002/advs.202520413 (PMC13073242; doi:10.1002/advs.202520413)
Supplement: Supplementary file 1 — Supporting File: advs73915‐sup‐0001‐SuppMat.docx. [file ADVS-13-e20413-s001.docx]

**Supporting Information**

**Ultrafast Multilevel Switching and Synaptic Behavior in a Planar Quantum Topological Memristor**

*Mamoon Ur Rashid^a^, Usman Safder^b^, Sobia Ali Khan^c^, Anh Tuan Pham^d^, Muhammad Sheeraz^a^, Nguyen-Hoang Dang^a^, Duy Le Thanh^e^, Zeeshan Tahir^f^, Faisal Maqbool^a^, Koo Hyun Chung^e^, Sunglae Cho^a^, Jungdae Kim^a^, and Yong Soo Kim^a^,**

1. Department of Semiconductor Engineering and Energy Harvest-Storage Research Center, University of Ulsan, Ulsan 44610, South Korea
2. School of Chemical and Bioprocess Engineering, University College Dublin (UCD), Dublin D04 V1W8, Ireland
3. New Energy Materials Research Institute, Korea Institute of Energy Technology (KENTECH), Naju 58330, South Korea
4. Division of Chemical and Material Metrology, Korea Research Institute of Standards and Science (KRISS), Daejeon 34113, South Korea
5. Department of Mechanical Engineering, University of Ulsan, Ulsan 44610, South Korea
6. Department of Physics and Chemistry, Daegu Gyeongbuk Institute of Science and Technology (DGIST), Daegu 42988, South Korea

Corresponding Author

* Email (Y.S. Kim): [yskim2@ulsan.ac.kr](mailto:yskim2@ulsan.ac.kr)

**Note S1.** **Conceptual illustration of a quantum topological insulator:**

Topological insulators (TIs) are unique class of quantum materials with layered 2D and 3D structures, characterized by metallic surface conduction and an insulating bulk. Figure S1a schematically illustrates the 3D layered structure of Bi_2_Te_3_ TI with three quintuple layers (QLs) separated by van der Waals gap. These weak interlayer bonds enable mechanical exfoliation, facilitating the integration of atomically thin QLs into nano electronic. TIs exhibit topologically protected surface states with spin-momentum locking and high carrier mobility enabling ultrafast and low-energy switching making it ideal to mimic biological synapses. Unlike conventional complementary metal-oxide-semiconductor systems limited by material constraints, TIs promise low-power and high-speed essential for next-generation neuromorphic architectures.^[1−3]^ Figure S1b demonstrates incorporation of quantum TIs within vertical and planar device layouts, where vertical design limits the exploitation of TI surface states due to buried interfaces, slower switching, and fabrication complexity.^[4−7]^ In contrast, the planar geometry directly exposes the conductive TI surface, offering, i) fast switching speed and low power consumption owing to direct surface interaction, ii) simplified device fabrication, and iii) enhanced versatility, supporting multi-terminal access open to optical stimuli. This direct utilization of robust, scatter-resistant surface conduction in planar architecture enhances the switching speed and energy efficiencies which are essential for unlocking the full potential of TIs in next-generation spintronic, neuromorphic, and optoelectronic devices.


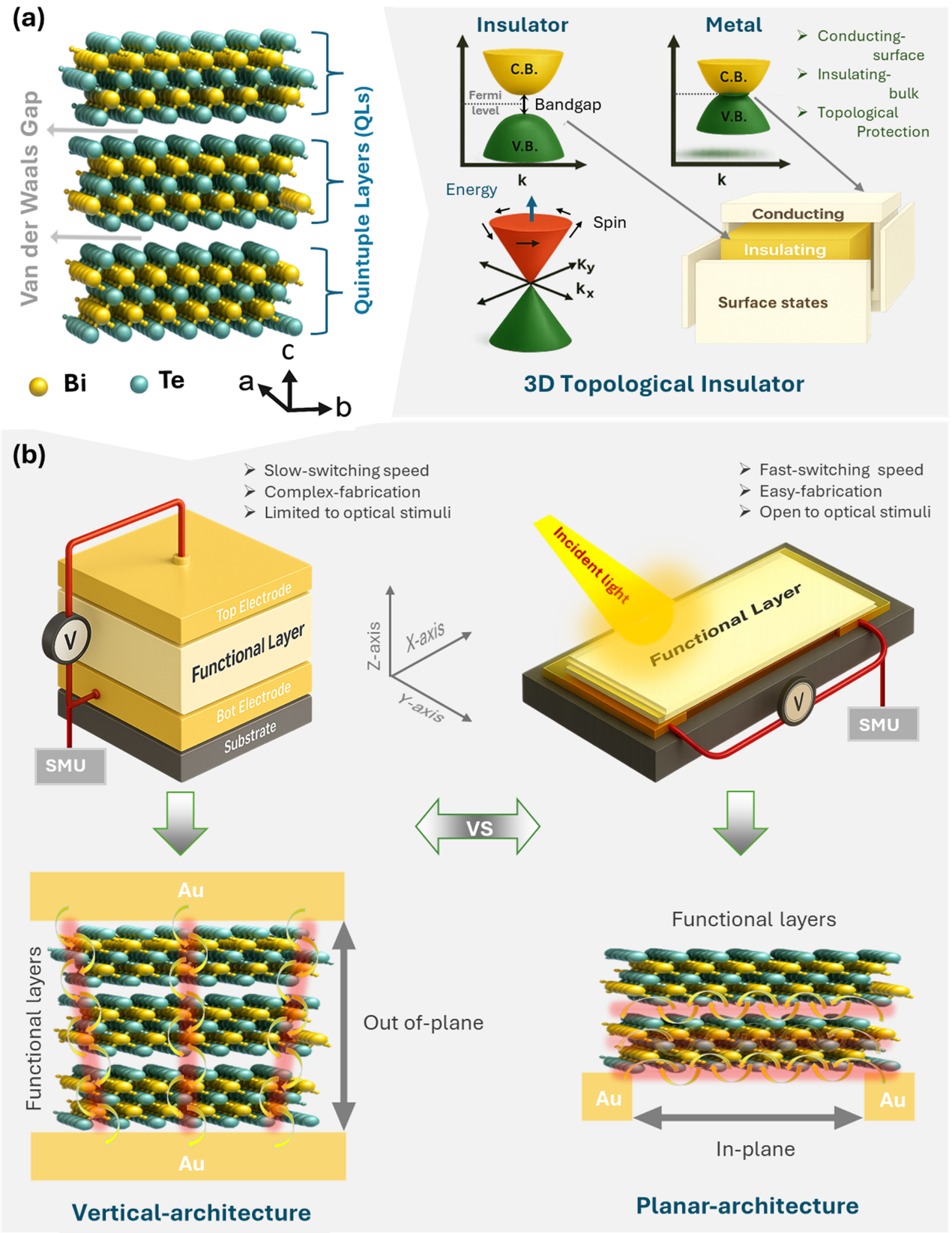


**Figure S1**. Conceptual illustration of a quantum topological insulator (QTI) and its integration into vertical and planar device architectures. (a) The 3D crystal structure of Bi_2_Te_3_ exhibits van der Waals gaps that separate each quintuple layer (QL), facilitating easy exfoliation. The right panel schematically explains the trademark feature of topological insulators, i.e., materials with conductive surfaces and insulating bulk or interiors. (b) Schematic comparison of vertical and planar device architectures. In contrast to vertical structure, the planar device directly harnesses the topological surface states owing to its peculiar in-plane architecture, thus offering relatively fast switching, along with other superiorities such as facile fabrication, and easy access for multi-terminal configurations, crucial for maximizing the functional potential of QTIs for synaptic applications.

**Note S2. Material growth:**

Single crystalline bismuth telluride (Bi_2_Te_3_) was synthesized using the temperature gradient method as illustrated schematically in left panel of Figure S2.^[8,9]^ The right panel demonstrates stepwise growth conditions of single crystals formation. Initially, high-purity (5N) Bi and Te powders were mixed in a 1:1.5 ratio and sealed in a quartz ampoule under a vacuum of less than 10^-4^ Torr. The ampoule was heated to 650 °C at 15 °C per hour and maintained for 16 hours to form a homogeneous molten Bi-Te mixture. Subsequently, the temperature was reduced slowly at 1 °C per hour to 500 °C, passing the Bi_2_Te_3_ melting point at 586 °C, before rapidly cooling down to room temperature. Note that it is crucial to maintain a cooling rate of 1 °C per hour across the melting point for effective single crystal growth. The controlled cooling ensures a uniform solidification rate throughout the crystal, facilitating the fabrication of high-quality single crystals.


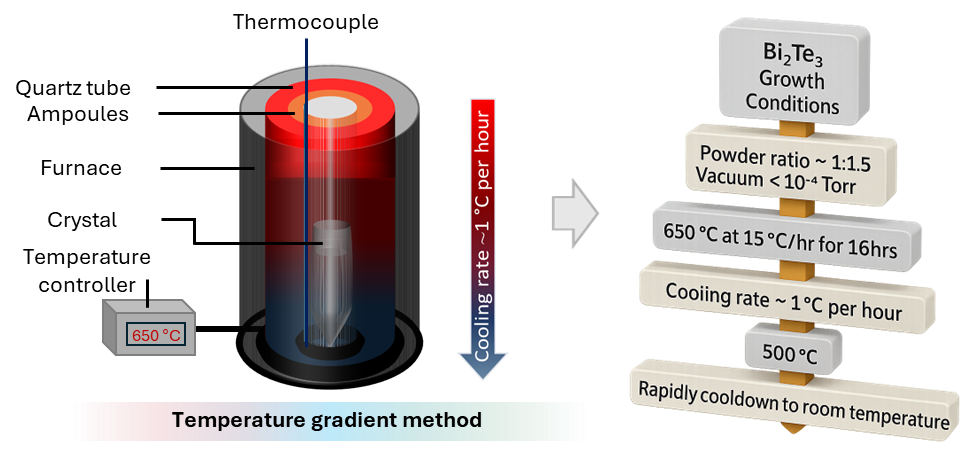


**Figure S2.** Stepwise synthesis procedure of bulk Bi_2_Te_3_ single crystals. The left and right panel presents schematic illustration of temperature gradient method and a sequence of all synthesis conditions respectively.

**
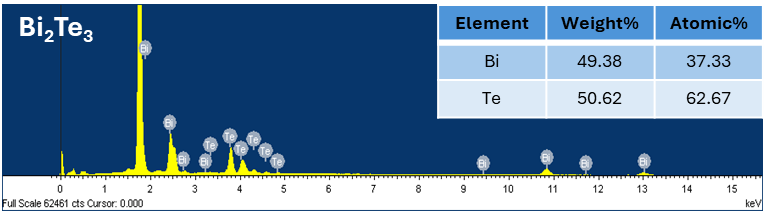
**

**Figure S3.** Energy-dispersive X-ray spectroscopy (EDS) spectrum of Bi_2_Te_3_ sample confirming the elemental composition. The spectrum exhibits prominent peaks corresponding to bismuth (Bi) and tellurium (Te), consistent with the stoichiometry of Bi_2_Te_3_. Quantitative analysis reveals a weight percentage of 49.38% for Bi and 50.62% for Te, corresponding to atomic percentages of 37.33% and 62.67%, respectively, indicating a near-stoichiometric composition.^[10,11]^

**
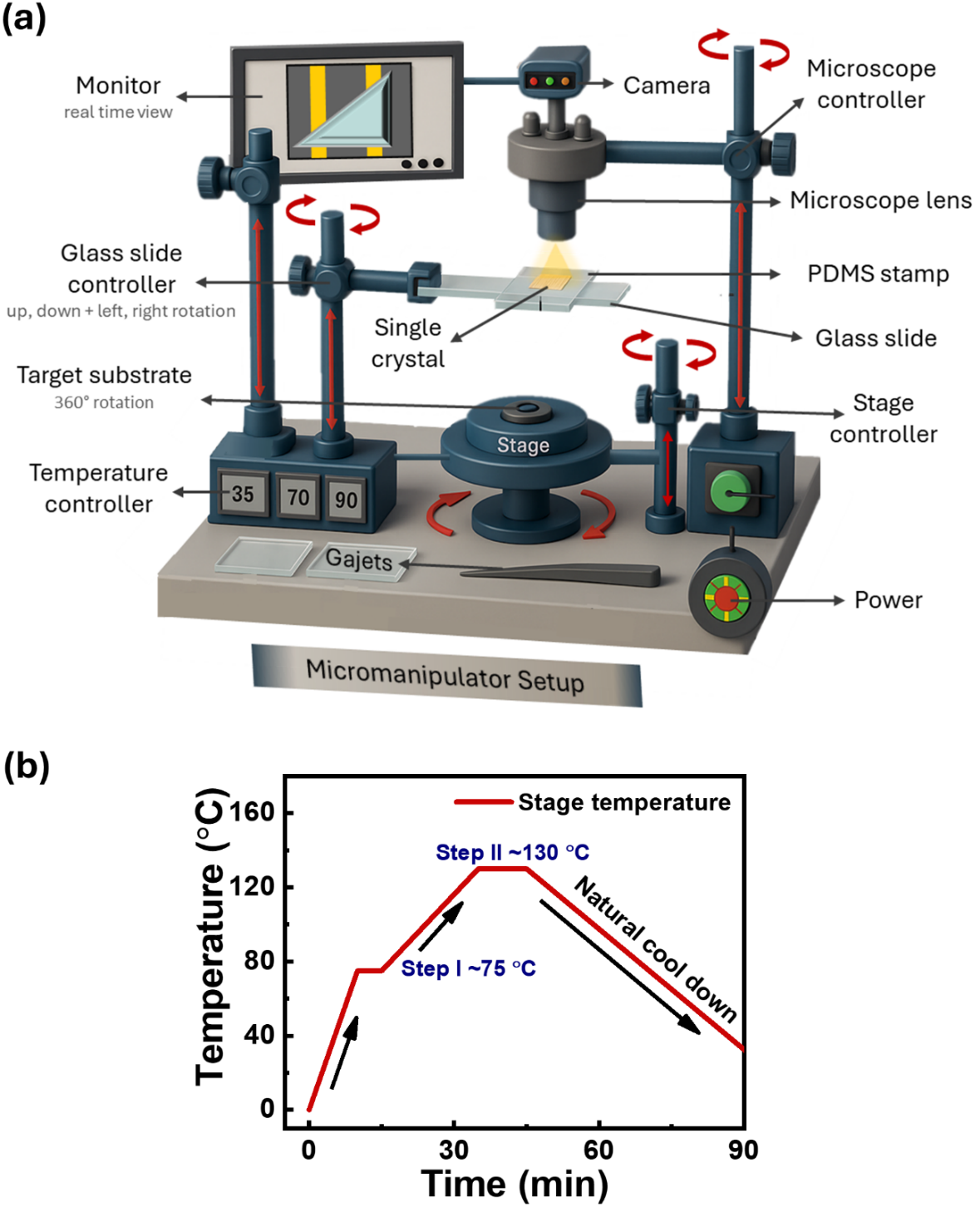
**

**Figure S4.** Homebuilt micromanipulator working procedure and annealing temperature profile. (a) Schematic illustration of our homebuilt micromanipulator setup, highlighting key components used for precise alignment during clean transfer of thin layers from polydimethylsiloxane (PDMS) onto top surface of prepatterned electrodes on the substrate. The system includes four manually controlled mechanical arms: (i) Glass slide controller, (ii) Stage controller, (iii) Microscope controller and (iv) Temperature controller, which together enable development of the final device. This process begins by placing a mechanically exfoliated crystal onto a transparent PDMS stamp mounted on a glass slide, which is then fixed upside down on the glass slide controller. The target substrate (where the crystal will be transferred) is securely positioned on the stage and precisely adjusted using the stage controller. Once the crystal and substrate are aligned using the microscope, glass slide, and stage controllers, the temperature controller is activated to initiate the transfer**.** (b) Real-time temperature vs time plot showing the thermal profile applied to the stage during the dry transfer process. This temperature ensures firm contact between the Bi_2_Te_3_ crystal and the prepatterned Au electrodes, allowing the PDMS to detach cleanly.


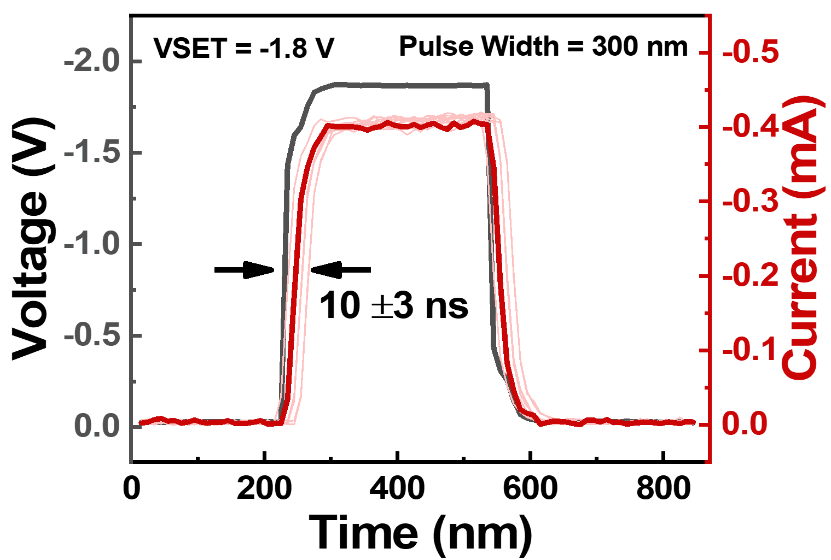


**Figure S5**. Switching dynamics of planar quantum topological memristor (PQTM). Transient voltage (black) and current (red) response of our planar Bi_2_Te_3_ PQTM under a 300 ns SET pulse ($V_{\mathrm{SET}}=-1.8$ V), demonstrating a rapid SET transition with a switching time of approximately $10$ns. This sharp transition, rather than a gradual exponential decay, indicates a genuine resistive switching event rather than a simple RC transient response.


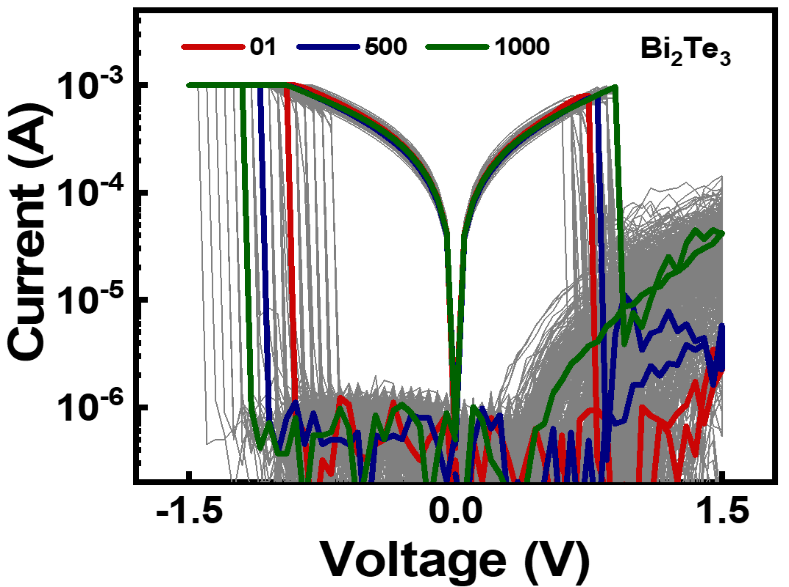


**Figure S6**. Stability test of PQTM. The semi-logarithmic *I-V* curves of PQTM demonstrate stable performance over 1000 consecutive DC cycles, maintaining consistent trajectories in both ON and OFF states matching well with Figure 3b distributions.


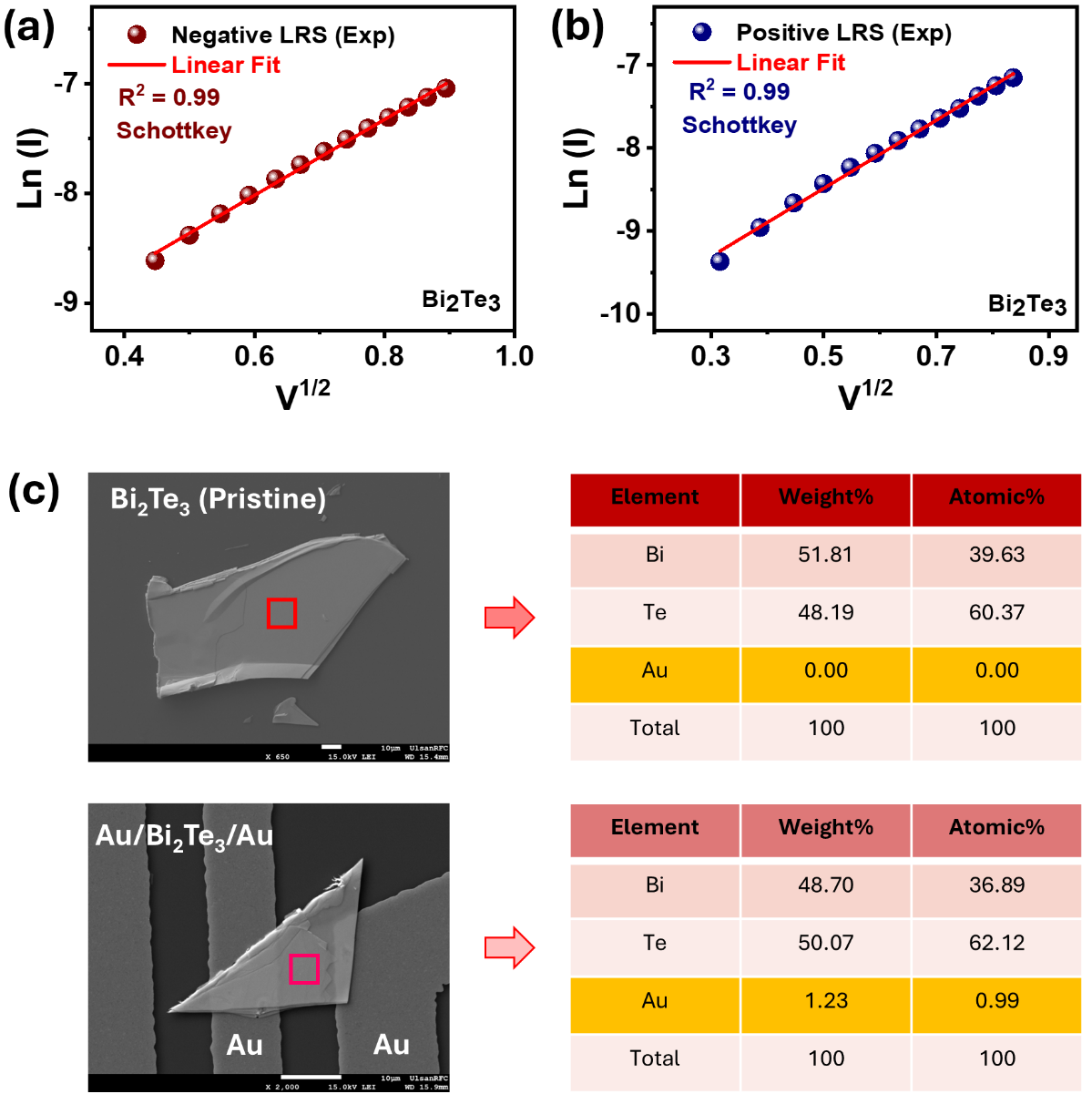


**Figure S7.** Conduction mechanism. (a, b) illustrate the possible switching behavior of Bi_2_Te_3_ based on *I-V* characteristics, analyzed through conduction mechanism fitting using Ln(*I*) vs *V*¹ᐟ² plots. At both bias low resistive states in *I-V* curve presenting the Schottky emission which belongs to electrode limited conduction mechanism.^[12]^ When a bias is applied across the electrodes, electrochemical reactions initiate Au ions to migrate into the active layer. These ions create traps which then help the movement of electrons and effectively modify the material's electrical conductivity. Au ions diffusion in Bi_2_Te_3_ is probably due to the lower bond strength in Bi-Te, and the large size of the Te atom,^[13,14]^ which originates the creation of a denser trap network to facilitate electron transportation and shows abrupt resistive switching. These results indicate that the cumulative effect of Au diffusion and topological surface conductance contributes to high-speed resistive switching. (c) A set of SEM images of pristine Bi_2_Te_3_ layers on SiO_2_/Si and the PQTM device, accompanied by their EDX tables. This verifies the clean transfer and provides the atomic and weight compositions from the highlighted regions. In the pristine sample, no Au signals were detected, confirming the absence of gold diffusion. In contrast, EDX analysis of the Au/Bi_2_Te_3_/Au device reveals the presence of Au content in the active region, indicating gold diffusion into the Bi_2_Te_3_ layer in the support of conduction mechanism as explain earlier.


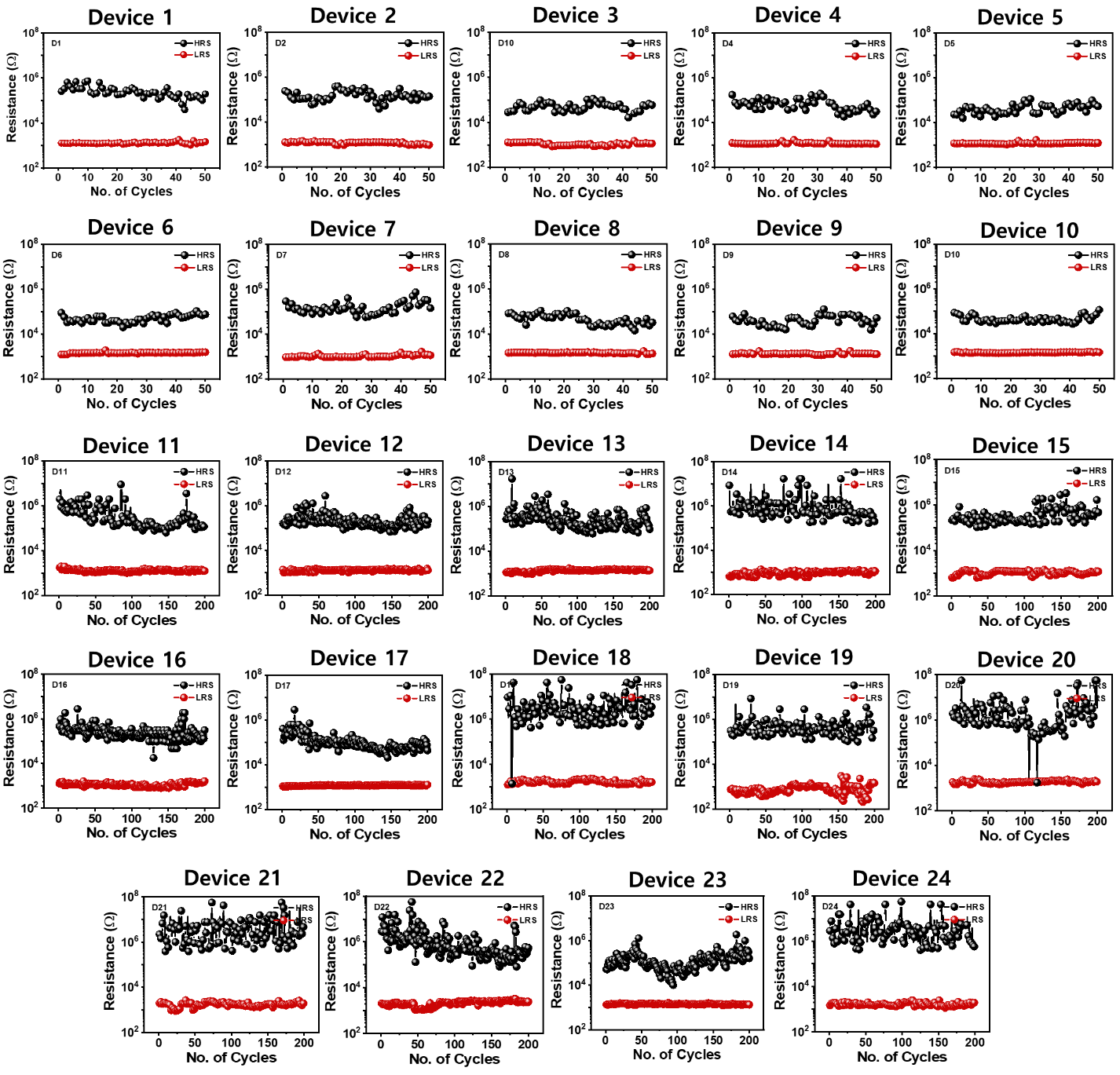


**Figure S8.** Reproducibility test of PQTMs. Device-to-device reproducibility is a critical factor in the development of reliable memory devices. To evaluate this, we analyzed the *I*-*V* characteristics of 24 independent Bi_2_Te_3_-based planar memristors fabricated under same conditions. The devices were tested over 50 (D1 to D10) and 200 (D11 to D24) consecutive switching cycles, all exhibiting bipolar resistive switching behavior. The high-resistance state (HRS, black) and low-resistance state (LRS, red) were extracted at a read voltage of −0.2 V. All devices show stable, forming-free bipolar switching with a reliable separation between HRS and LRS. The switching behavior of these devices closely matches that of the original device, confirming that the performance is reproducible and not device specific. Although minor cycle-to-cycle variations were noted particularly in the HRS, which is common in typical memristors. Thus, all devices maintain a clear memory window throughout the endurance measurements with no evidence of failure or state degradation.


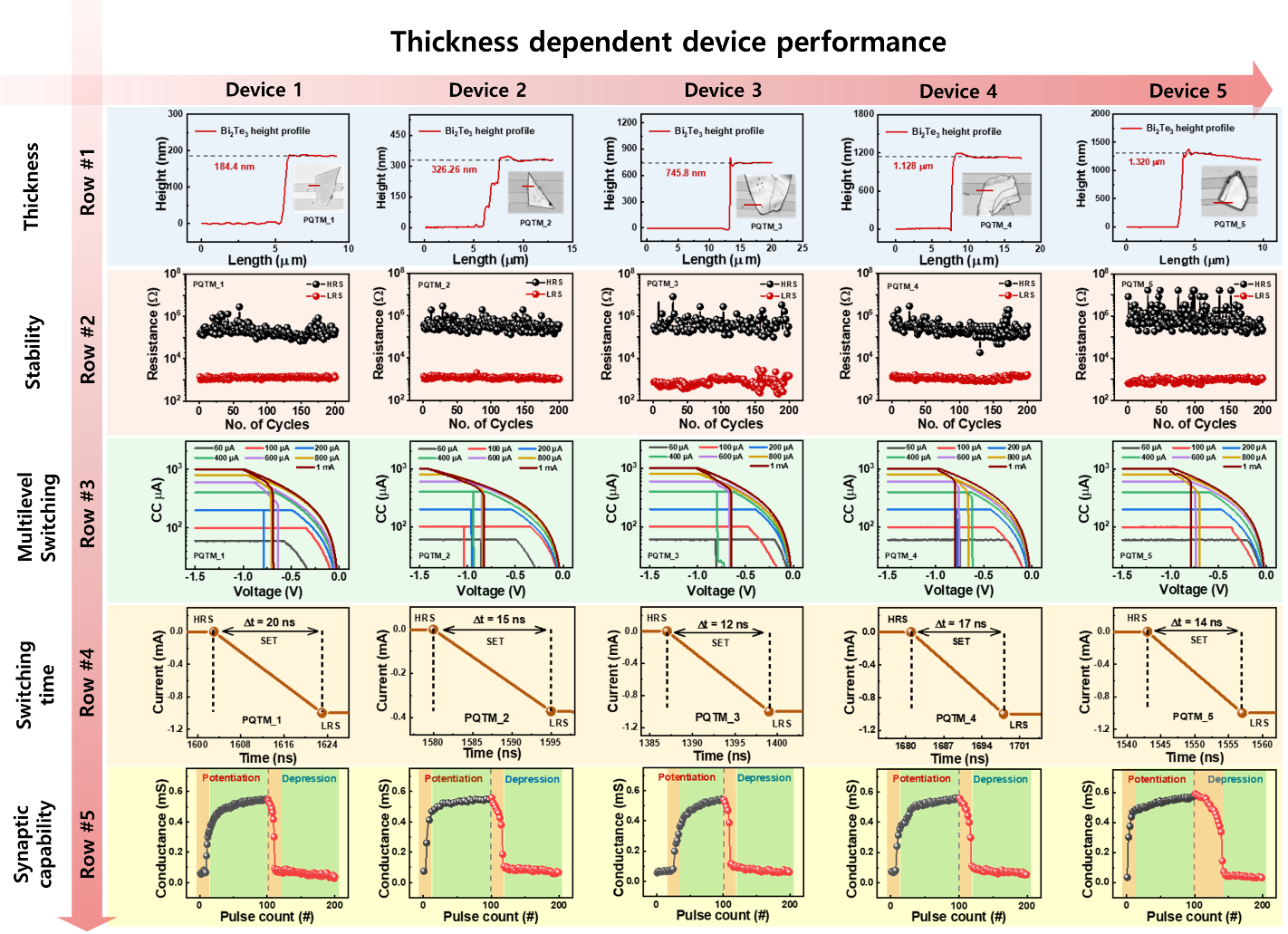


**Figure S9.** Thickness-dependent performance of PQTMs. Devices are arranged from left to right in order of increasing thickness. From top to bottom, each column illustrates the performance of an individual device, while each row compares five devices with different thicknesses. This comparison reveals nearly identical performance in terms of stability characteristics, multilevel switching behavior, switching time, and synaptic capabilities, demonstrating the device performances independent of Bi_2_Te_3_ film thickness. Row #1 represents line profiles revealing devices of various thickness ranging from 184.4 nm to 1320 nm, measured via confocal microscope (optical image in inset). Row #2 highlights the endurance of these devices up to 200 cycles, each exhibiting a consistent ON/OFF ratio with minor fluctuations in HRS. Similarly, row #3, row #4 and row #5 also showcases consistency with respect to multilevel switching, switching time and synaptic capability (potentiation and depression) respectively, matching the results in the main text, Thus, this thickness independence further strengthens and support surface-assisted switching, consistent with transport facilitated by TSS in PQTMs (Figure 2f).

**Table S1.** Thickness-dependent performances of PQTMs.

*
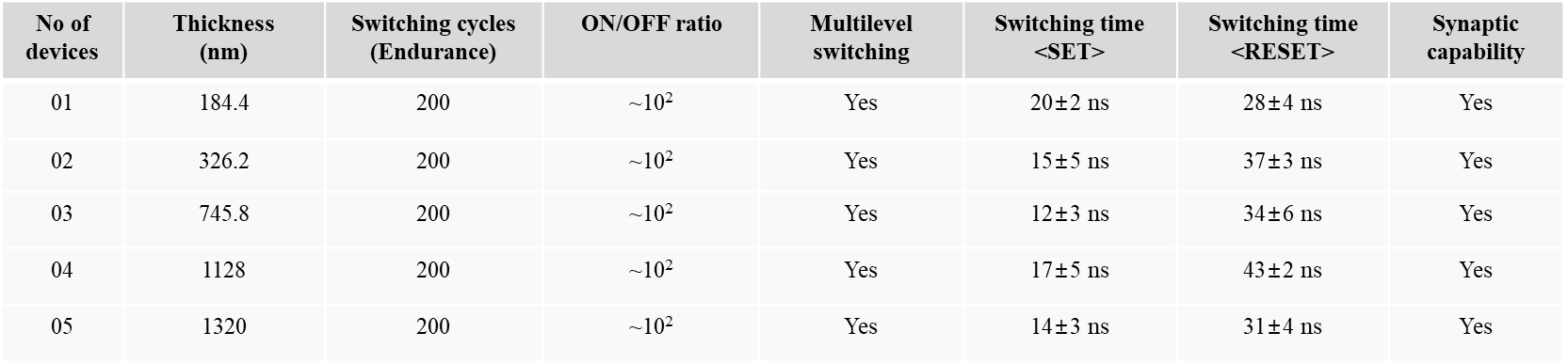
*

**Table S1****.** It was observed that all devices with various thicknesses represent nearly identical performance in all aspects, as summarized in the above table. These results indicate that the active switching pathway is confined to the surface or electrode interface rather than the bulk of the material, revealing the thickness independence of Bi_2_Te_3_ films. This thickness independence further strengthens the case for surface-assisted switching, consistent with transport facilitated by TSS in PQTMs.

**Note S3. Energy efficiency calculations**

Power and energy consumption were calculated using these standard equations,^[15]^

$P_{i}=V_{r}\times I_{i}$ (A1)

$E_{i}=V_{P}\times I_{i}\times t$ (A2)

where $V$*_r_* = 0.2 V is the read voltage for each synaptic event, *I_i_* is the current for the *i^th^* pulse measured at the corresponding read voltage, $V_{P}$ = 0.5 V is the programming pulse amplitude, and *t* = 1 ms is the time duration (pulse width). By examining the pulse dataset, the average power and energy consumption per synaptic event was determined to be approximately 5.8 µW, and 14.5 nJ, respectively.

**Note S4. Simulation details**

These vectors (*𝑥_1_, 𝑥_2_*) were processed as one-dimensional signals and reshaped into input tensors of shape (batch size, 1, 2) to serve as inputs to a lightweight 1D convolutional neural network (CNN). The architecture consists of two consecutive 1D convolutional layers with ReLU activations (8 and 16 filters, kernel size = 1), followed by flattening and two fully connected layers (32 and 16 neurons), and a final output layer with 2 neurons for binary classification as shown in Table S1. Training was performed using the Adam optimizer with a learning rate of 1×10^-3^, a batch size of 32 for 20 epochs. Categorical cross-entropy was used as the loss function. Quantized and binarized versions of this network were also evaluated to assess inference robustness under precision-constrained deployments. To benchmark the network architecture against standard image recognition tasks, the MNIST dataset was employed. The dataset contains 70,000 grayscale images of handwritten digits (0-9), each sized 28×28 pixels. The training and test sets comprise 60,000 and 10,000 images, respectively. Images were normalized and reshaped into tensors of shape (batch size, 1, 28, 28), compatible with the LeNet architecture.

**Table S*2***. LeNet CNN architecture for neuromorphic device classification.

| **Layer** | **Type** | **Input Shape** | **Kernel** | **Output Shape** | **Activation** |
| --- | --- | --- | --- | --- | --- |
| Input | Input Layer | (batch, 1, 2) | — | (batch, 1, 2) | — |
| Conv1 | 1D Convolutional | (batch, 1, 2) | 8 filters × 1 | (batch, 8, 2) | ReLU |
| Conv2 | 1D Convolutional | (batch, 8, 2) | 16 filters × 1 | (batch, 16, 2) | ReLU |
| Flatten | Reshape | (batch, 16, 2) | — | (batch, 32) | — |
| FC1 | Fully Connected | (batch, 32) | 32 units | (batch, 32) | ReLU |
| FC2 | Fully Connected | (batch, 32) | 16 units | (batch, 16) | ReLU |
| Output (FC3) | Fully Connected | (batch, 16) | 2 units | (batch, 2) | Softmax* |

**Softmax activation is applied during inference to compute class probabilities for depression- vs potentiation-dominant responses.*

**Table S2**. Simulation architecture comprising two consecutive 1D convolutional layers with ReLU activations, followed by a flattening layer, two fully connected layers, and a final output layer with two neurons for binary classification.


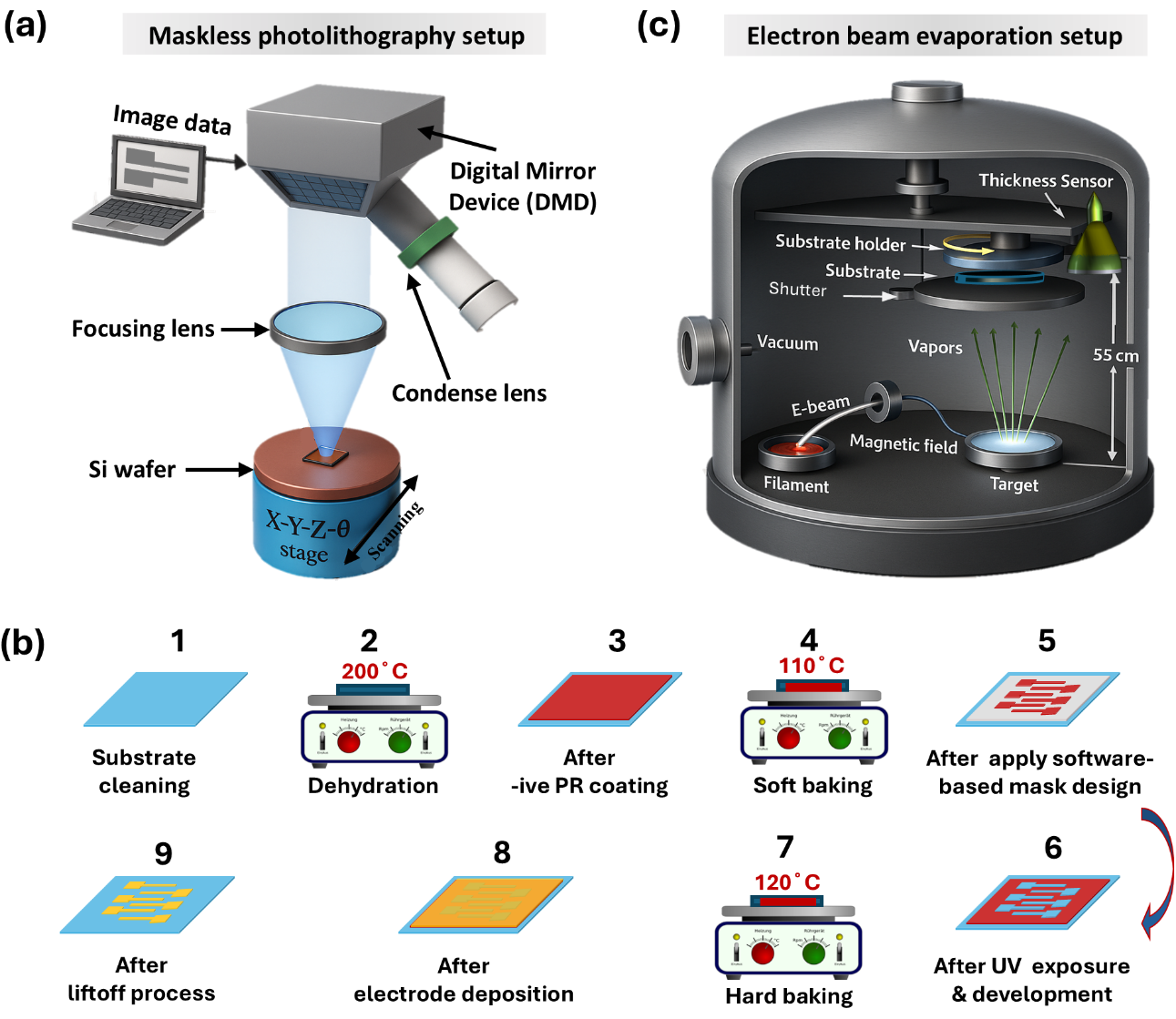


**Figure S10.** Maskless photolithography synthesis procedure and electron beam electrode deposition. (a) Maskless photolithography operates on principles like conventional photolithography but offers key advantages. By directly writing patterns from computer-generated data, it eliminates the need for physical masks, reducing fabrication time, cost, and enabling rapid design iteration. This approach enhances flexibility, resolution, and feature scalability while minimizing material waste and simplifying the process flow. We employed this technique to define electrode patterns for planar memristor devices, as illustrated below. (b) Schematically illustrates the lithography process in detail, beginning with substrate cleaning and dehydration, followed by coating with a light-sensitive photoresist (negative PR). After soft-baking, a computer-generated mask pattern is aligned over the PR-coated substrate and exposed to UV light, which alters the solubility of the exposed regions based on the type of photoresist. The developed substrate is then hard baked to stabilize the pattern. As shown in (c) Au is selectively deposited using electron-beam evaporation on the patterned substrate using the similar conditions in our previous report.^[16]^ Finally, a lift-off process is performed to define the electrode patterns for subsequent Bi_2_Te_3_ crystal transfer.

**References.**

[1] D. S. Assi, H. Huang, V. Karthikeyan, V. C. S. Theja, M. M. D. Souza, N. Xi, W. J. Li, V. A. L. Roy, Adv. Sci.10 **2023,** 24, 2300791.

[2] Z. Wan, Q. Zhang, F. Hu, Y. Dong, R Li, L. Hu, Y. Xie, Z. Yue, X. Chen, M. Gu, Adv. Opt. Mater. **2023,** 11, 2, 2201852.

[3] P. Roushan, J. Seo, C. V. Parker, Y. S. Hor, D. Hsieh, D. Qian, A. Richardela, M. Z. Hasan, R. J. Cava. A. Yazdani, Nature **2009,** 460, 7259, 1106-1109.

[4] Y. Zuo, H. Lin, J. Guo, Y. Yuan, H. He, Y. Li, Y. Xiao, X. Li, K. Zhu, T. wang, X. Jung, C. Wen, M. Lanza, Advanced Electronic Materials. **2020,** 6, 3, 1901226.

[6] Y. Xiao, B. Jiang, Z. Zhang, S. Ke, Y. Jin, X. Wen, C. Ye, Technol. Adv. Mat. **2023,** 24, 1, 2162323.

[7] A. Kiazadeh, H. L. Gomes, A. R. D. Costa, P. Rocha, Q. Chen, J. A. Moreira, D. M. D. Leeuw, S. C. J. Meskers, MRS Online Proceedings Library (OPL), **2011,** 1337.

[8] Duong. A. T, Nguyen. T. T. H, Nguyen. T. M. H, Pham. A. T, Dinh T. M. Hao, Sungale. C, VNU Journal of Science: Mathematics-Physics **2019,** 35, 1, 41-46.

[9] Van. Q. N, Jundae. Kim, Sunglae. Cho , J. Korean Phys. Soc. **2018,** 72, 8, 841-857.

[10] A. Raj, A. Kumar, R. Kumar, R. Kumar, R. Chnadra, J. Mater. Sci.: Mater. Electron. **2024,** 35, 7, 468

[11] N. D. Desai, V. L. Patil, S.S. Patil, P. S. Patil, ChemistrySelect, **2022,** 7, 44, e202202965.

[12] F. C. Chiu, Adv. Mater. Sci. Eng. **2014,** 1, 578168.

[13] A. Soni, Z. Yanyuan, Y. Ligen, M. K. K. Aik, M. S. Dresselhaus, Q. Xiong, Nano Lett. **2012,** 12, 3, 1203-1209.

[14] Y. N. Nguyen, I. Son, Mater. Chem. Phys. **2022,** 292, 126813.

[15] D. S. Jeong, K. M. Kim, S. Kim, B. J. Choi, C. S. Hwang, Adv. Electron. Mater. **2016,** 2, 9, 1600090.

[16] M. U. Rashid, S. A. Khan, F. Ghafoor, J. S. Bae, Y. C. Park, M. Sheeraz, Z. Tahir, C. T. Le, Y. S. Kim, Curr. Appl. Phys. **2024,** 64, 16-24.
